# Similarity measure and domain adaptation in multiple mixture model clustering: An application to image processing

**DOI:** 10.1371/journal.pone.0180307

**Published:** 2017-07-07

**Authors:** Siow Hoo Leong, Seng Huat Ong

**Affiliations:** 1 Faculty of Computer and Mathematical Sciences, Universiti Teknologi MARA, Kota Samarahan, Sarawak, Malaysia; 2 Institute of Mathematical Sciences, University of Malaya, Kuala Lumpur, Malaysia; University of North Carolina at Chapel Hill, UNITED STATES

## Abstract

This paper considers three crucial issues in processing scaled down image, the representation of partial image, similarity measure and domain adaptation. Two Gaussian mixture model based algorithms are proposed to effectively preserve image details and avoids image degradation. Multiple partial images are clustered separately through Gaussian mixture model clustering with a scan and select procedure to enhance the inclusion of small image details. The local image features, represented by maximum likelihood estimates of the mixture components, are classified by using the modified Bayes factor (MBF) as a similarity measure. The detection of novel local features from MBF will suggest domain adaptation, which is changing the number of components of the Gaussian mixture model. The performance of the proposed algorithms are evaluated with simulated data and real images and it is shown to perform much better than existing Gaussian mixture model based algorithms in reproducing images with higher structural similarity index.

## 1 Introduction

The processing of an image as a whole becomes more challenging with the increase in the image data size. In a lot of the applications of image analysis, it is not feasible to process an entire image of a large size. The most common approach to addressing this problem is to scale down the data size so that the computational complexity can be reduced. There are popular methods used for scaling down image data size: i) sampling–start with a subset of the image data, [1–4], and ii) partition into blocks–divide an image into *m* x *n* blocks, [5–10]. Although these methods are simple, they have been developed into popular techniques, for examples, bag-of-features [11], block based compressed sensing [12].

The basic notion of scaling down data through sampling is to apply an extended clustering scheme [4] where the clustering algorithm is first performed on a manageable sample of the whole data set, and then the results are extended to classify the remaining data. The main drawback of this simple method is that the number of clusters captured in the training sample may not represent all the clusters in the whole data set. In other words, the source domain and the target domain are different. Without domain adaptation during the extension to classify the whole data set, it tends to miss out small but important information. The shortcoming of overlooking small localized variation of image components may not be significantly reflected in the global error distortion measures such as mean square error (MSE) or signal to noise ratio, but it is an important issue to be addressed especially in medical imaging as often there are only subtle differences in visual features between the normal and pathological images [13]. In the Gaussian mixture model (GMM) framework, some works have been proposed to improve the clustering result of the selected sample. For example, algorithms for splitting clusters based on statistical tests have been proposed to improve the accuracy of the clusters captured by the training sample [14–15]. However, these algorithms lead to discovery of false clusters. Variants of the expectation and maximization (EM) algorithm have been proposed to improve the capture of small clusters [16–17] and the identification of overlapping clusters [18–19] in the training sample. However, this does not solve the problem of lack of representativeness of the training sample. [1] improves the unstable results of the sampling based algorithm by selecting several best models [20–21] based on the training sample data, and run several EM steps on the full data set to select the final best model. Their recommendation of using multiple samples as unsupervised training sets motivates the development of the first algorithm in this paper, FlexClustS. The proposed sampling based GMM algorithm performs domain adaptation among the clustering results from multiple samples, and therefore improves the existing algorithms, especially [20–21], from three aspects: (i) recovers clusters that have not been identified in the training sample, (ii) recovers small but important clusters, (iii) preserves image features better, and (iv) does not unrealistically pre-define the number of clusters in the whole data set.

On the other hand, algorithms that work on image data divided into blocks normally consist of two phases. In the first phase, each block of the image data is compressed or summarized and represented by descriptors (or prototypes) of features. Then, the collection of descriptors from all the blocks are incorporated based on a particular similarity measure such as Euclidean, Mahalanobis or Manhattan distance. One of the most noticeable degradations of this method is blocking artifacts [22–23]. This happened when the local features from each block of image are processed independently without taking into account the information of the adjacent blocks, and it results in discontinuities in the block boundaries. In the existing GMM based algorithms, each block of data is normally summarized by *k-*means method, and each resulting subcluster is represented by a descriptor of triplet statistics (mean, variance and number of data points). Then, a variant of expectation and maximization (EM) algorithm is used to fit the descriptors from all the blocks of data into GMM [24–25]. There are shortcomings in these algorithms. First, using *k*-means as the partial image representation model does not capture well of the image features from each block if the pixel clusters are not spherical in shape. Second, GMM clustering based on variant of EM increases the computational complexity, especially if the cumulative number of descriptors from all the blocks is large. Therefore, this paper proposes the second algorithm based on multiple blocks of image data, FlexClustB, to improve the existing algorithms, especially [24–25] from two three aspects: (i) preserves image features better, (ii) reduces computational complexity during clustering of all descriptors by using similarity measure, and (iii) avoids blocking artifact.

This paper proposes two GMM based algorithms which are termed as FlexClustS (Flexible number of clusters–sampling based) and FlexClustB (Flexible number of clusters–block based). For ease of explanation in the following sections, FlexCustS and FlexClustB are grouped under FlexClust. The two algorithms are quite similar except for the method used to scale down the data size. A brief description of the two proposed algorithms is given as follows: First the image data is scaled down by dividing it into multiple samples or *m* x *n* blocks for FlexClustS and FlexCLustB respectively. A scan through and selection procedure is proposed for initialization of the GMM, and each sample or block of the image is represented by a GMM. The idea of scan through and selection procedure is adapted from [26–27] to isolate the small details of the image and over represent these pixels to increase the chance of their detection. GMM is chosen in this paper because it has been proven to be effective for patterns representation and it preserves the image features well as exemplified in many applications: classification of and 12-lead electrocardiogram (ECG) [28]; segmentation of image [29] and brain magnetic resonance images [30]. This paper proposes to use the maximum likelihood estimates (MLEs) of the GMM as the local image descriptor for each sample or block. Next, the descriptors of MLEs resulting from multiple GMM clustering will be aggregated into a compact representation of the entire image by a proposed mixture model distribution. This is done by considering the image representation of one of the samples or blocks as source domain, and the remaining being representation of samples or blocks as target domain that are to be classified. The classification is based on a proposed pairwise similarity measure known as modified Bayes factor (MBF), which is an adapted Bayesian model selection criterion. If the MBF suggests that any descriptor has novel local features, the proposed model is updated by allowing domain adaptation to change the number of mixture components.

The main contributions of this paper are summarized as follows:

1. The introduction of two algorithms, FlexClustS and FlexClustB, to work on scaled down image data more effectively in preserving the image details and avoids the problem of blocking artifacts.
2. Propose the Gaussian mixture model with a scan through and selection procedure for feature extraction, which enhances the possibility of the detection of small details of the image.
3. Propose the modified Bayes factor for similarity measure, which makes use of the partial image descriptors, and detects novel local image features for domain adaptation
4. A mixture model distribution for compact representation of the entire image, which takes care of domain adaptation when classifying the local image descriptors obtained from samples or blocks of image.

The remainder of paper is organized as follows. Section 2 briefly reviews the theoretical background of the Gaussian mixture models related to the proposed algorithm. Section 3 describes the detail of the FlexClustS and FlexClustB algorithms. Section 4 presents the results on simulated data and application to real images. Finally, the discussion and conclusion are presented in Sections 5.

## 2 Theoretical background

In this section, we describe the Gaussian mixture model since it is closely related to the proposed algorithm. From this section onward, the components of the mixture model also refer to the groups, clusters or classes of pixels.

### 2.1 Gaussian mixture model for clustering

In this paper, the Gaussian mixture model (GMM), with improvement in initialization, is used to compress the samples or blocks of image through clustering. Performing clustering via mixture models not only has the advantage of having a means of estimating the parameters of the model by employing the expectation-maximization (EM) algorithm, but also helps to determine the number of clusters through the comparison of the Bayesian Information Criterion (BIC) [31].

In mixture model clustering of image data, the *d*-dimensional random pixels of size *n*, **x**_1_, …, **x**_*n*_, are assumed to have been generated from a mixture of a finite number, say *G*, of underlying probability distributions. The mixture density for each **x**_*i*_ is expressed as

$$f(x_{i}|\Psi )=\sum_{k=1}^{G}\pi_{k}f_{k}(x_{i}|\theta_{k}),\;i=1,2,\ldots ,n$$ (1)

where *π*_*k*_ is the non negative mixture proportion for the *k*th component which satisfies Σ *π*_*k*_ = 1, and **Ψ =** (*π*_*1*_, …, *π*_*G*_, *θ*_*1*_, …, *θ*_*G*_)is the vector of all the unknown parameters. In GMM, the parameter **θ**_*k*_ consists of a mean vector **μ**_*k*_ and a covariance matrix **Σ**_*k*_, and the density has the form

$$f_{k}(x_{i}|\theta_{k})=\varphi_{k}(x_{i}|\mu_{k},\Sigma_{k})={\left(2\pi \right)}^{-p/2}|\Sigma_{k}{|}^{-1/2}\exp\left\{1/2{\left(x_{i}-\mu_{k}\right)}^{T}\Sigma_{k}^{-1}\left(x_{i}-\mu_{k}\right)\right\},$$ (2)

where |**Σ**_*k*_| is the determinant of the covariance matrix.

The MLE of parameters of the mixture model can be estimated iteratively by applying the EM algorithm [32]. In clustering, the EM algorithm for clustering is a general approach to maximize the likelihood function in the presence of a set of unobservable group-indicators **z**_1_, …, **z**_*n*_ which are treated as incomplete data. Each of these indicators has the form **z**_*i*_ = (z_*i*1_, …, z_*iG*_) with z_*ik*_ = 1 if **x**_*i*_ belongs to group *k*, otherwise z_*ik*_ = 0. Therefore, the complete data log likelihood of GMM is given by

$$\log L(\Psi ,z|x)=\sum_{i=1}^{n}\sum_{k=1}^{G}z_{ik}\log[\pi_{k}\varphi_{k}(x_{i},\mu_{k},\Sigma_{k})].$$ (3)

An iteration of EM algorithm for GMM is as follows: in the E-step of the *t*th iteration, calculate the conditional probabilities, z_*ik*_, that **x**_*i*_ arises from the *k*th mixture components for the current value of the mixture parameters as given by

$$z_{ik}^{\left(t\right)}=\frac{\pi_{k}^{\left(t\right)}\varphi_{k}(x_{i}|\mu_{k}^{\left(t\right)},\Sigma_{k}^{\left(t\right)})}{\sum_{k=1}^{G}\pi_{k}^{\left(t\right)}\varphi_{k}(x_{i}|\mu_{k}^{\left(t\right)},\Sigma_{k}^{\left(t\right)})},$$ (4)

while the M-step of the (*t*+ 1)th iteration involves update of mixture parameters estimates, *π*_*k*_, **μ**_*k*_, and **Σ**_*k*_, maximizing Eq (3) by substituting the values of z_*ik*_^(*t*)^ computed from Eq (4) as follows:

$$\begin{array}{l} n_{k}^{\left(t+1\right)}=\sum_{i=1}^{n}z_{ik}^{\left(t\right)}\\ \pi_{i}^{\left(t+1\right)}=n_{k}^{\left(t+1\right)}/n\\ \mu_{i}^{\left(t+1\right)}=\sum_{i=1}^{n}z_{ik}^{\left(t\right)}x_{i}\\ \Sigma_{k}^{\left(t+1\right)}=\{\sum_{i=1}^{n}z_{ik}^{\left(t\right)}(x_{i}-\mu_{i}^{\left(t+1\right)})(x_{i}-\mu_{i}^{\left(t+1\right)}{)}^{\prime }\}/n_{k}^{\left(t+1\right)}. \end{array}$$ (5)

Let the MLEs of the GMM be

$$\hat{\Psi }=({\hat{\pi }}_{k},{\hat{\mu }}_{k},{\hat{\Sigma }}_{k}),$$

for *k* = 1, 2, … *k*, the pixel **x**_*i*_ can be assigned to the component of the mixture with the highest estimated posterior probability where

$${\hat{z}}_{ig}=1,\;\mathrm{if}\;g=\arg\;\underset{k}{\max}\;{\hat{z}}_{ik};\;{\hat{z}}_{ig}=0,\;\mathrm{otherwise}.$$ (6)

One of the advantages of using mixture model clustering is that the model with the appropriate number of clusters or mixture components may be chosen by using the Bayesian Information Criterion (BIC) [33],

$$\mathrm{BIC}=-2\;\log L(\hat{\Psi }|x)+p\log n,$$ (7)

where *p* is the functionally independent parameters to be estimated in the MLEs of the GMM.The selected model is the one with the minimum BIC.

### 2.2 Gaussian mixture model for classification

When sampling is used for scaling down the data size for GMM based image processing, the common procedure is to perform unsupervised training through GMM clustering for the pixel sample as described in Section 2.1, and then use discriminant analysis to classify the remainder of the image pixels [20–21]. Basically, the GMM for classification or discriminant analysis applies one E-step to the remainder of the image data using the parameters obtained from the clustered sample. The posterior probability that a pixel **x**_*i*_ belong to the *k*th class is calculated by

$$P(x_{i}\in class\;k)=\frac{\pi_{k}\varphi_{k}(x_{i}|\mu_{k},\Sigma_{k})}{\sum_{k=1}^{G}\pi_{k}\varphi_{k}(x_{i}|\mu_{k},\Sigma_{k})},$$ (8)

and the pixel is classified to the class in which it has the highest posterior probability.

### 2.3 Gaussian mixture model for summarized data

Dividing an image into blocks is always followed by the compression step where the image data of each block is summarized by a specific set of quantities (prototype or descriptor). Gaussian mixture model for summarized data has been introduced by [24–25]. The basic notion is to perform a variant of EM algorithm for the descriptor of triplet statistics (mean, variance and number of data points) or sufficient statistics.

Assume that a data set has been summarized to a set of *m* descriptors of sufficient statistics (${\bar{x}}_{i}$, *S*_*i*_, *n*_*i*_), for *i* = 1, 2, …,*m*, where ${\bar{x}}_{i}$ and ***S***_*i*_ are the mean vector and covariance matrix of the summarized data points for descriptor *i*, and *n*_*i*_ is the number of data points. Then, the corresponding complete log likelihood for the prototype set is given by

$$\log\;L_{c}^{s}(\Psi |{\bar{x}}_{1},\ldots ,{\bar{x}}_{m},z)=\sum_{i=1}^{m}\sum_{k=1}^{g}z_{ik}n_{i}\log[\pi_{k}\varphi_{k}({\bar{x}}_{i};\mu_{k},\Sigma_{k})],$$ (9)

where **z** = (**z**’_1, …,_ **z**’_*m*_)’ denotes the component membership of the *m* descriptors.

The sufficient EM algorithm operates on the sufficient statistics to maximize the complete descriptor log likelihood in Eq (9) [25]. For the *t*th iteration, the mean vectors ${\bar{x}}_{i}$ are used in the E-step to calculate the expected component memberships z_*ik*_^(*t*)^ of the descriptors which are equal to their posterior probabilities

$$z_{ik}^{\left(t\right)}=\frac{\pi_{k}^{\left(t\right)}\varphi ({\bar{x}}_{i};\mu_{k}^{\left(t\right)},\Sigma_{k}^{\left(t\right)})}{\sum_{h=1}^{g}\pi_{h}^{\left(t\right)}\varphi ({\bar{x}}_{i};\mu_{h}^{\left(t\right)},\Sigma_{h}^{\left(t\right)})}.$$ (10)

In iteration *t*+1, the weights which reflect the descriptor sizes, *n*_*i*_ z_*ik*_^(*t*)^, are introduced. Therefore, the component means are calculated as the weighted sum of prototypes means given by

$$\mu_{k}^{\left(t+1\right)}=\sum_{i=1}^{m}n_{i}z_{ik}^{\left(t\right)}{\bar{x}}_{i}/\sum_{h=1}^{m}n_{h}z_{hk}^{\left(t\right)},$$ (11)

the component covariance matrices are calculated by decomposing into sum of the weighted between and within descriptor sum of squares and products matrices **B**_*SSP*,*k*_^(*t*)^ and **W**_*SSP*,*k*_^(*t*)^ respectively, given by

$$\begin{array}{l} \Sigma_{k}^{\left(t+1\right)}=\left[\sum_{i=1}^{m}n_{i}z_{ik}^{\left(t\right)}({\bar{x}}_{i}-\mu_{k}^{\left(t\right)})({\bar{x}}_{i}-\mu_{k}^{\left(t\right)})+\sum_{i=1}^{m}n_{i}z_{ik}^{\left(t\right)}S_{i}\right]/\sum_{h=1}^{m}n_{h}z_{hk}^{\left(t\right)}\\ \;=\left[B_{SSP,k}^{\left(t\right)}+W_{SSP,k}^{\left(t\right)}\right]/\sum_{h=1}^{m}n_{h}z_{hk}^{\left(t\right)}\\ \;=B_{k}^{\left(t\right)}+W_{k}^{\left(t\right)} \end{array},$$ (12)

and the mixing proportions are given by

$$\pi_{k}^{\left(t+1\right)}=\sum_{i=1}^{m}n_{i}z_{ik}^{\left(t\right)}/\sum_{i=1}^{m}n_{i},$$ (13)

for all the mixture components *k* = 1, …, *g*.

The number of mixture components is assessed by a variant of BIC given by

$$-2\;\log L^{s}(\hat{\Psi })+d\log n,$$ (14)

where the likelihood is sufficient likelihood from an approximation of the likelihood of the original data, *d* is the number of parameters to be estimated for the mixture, and *n* is the number of single observations.

## 3 The FlexClust algorithms

**Fig 1:**
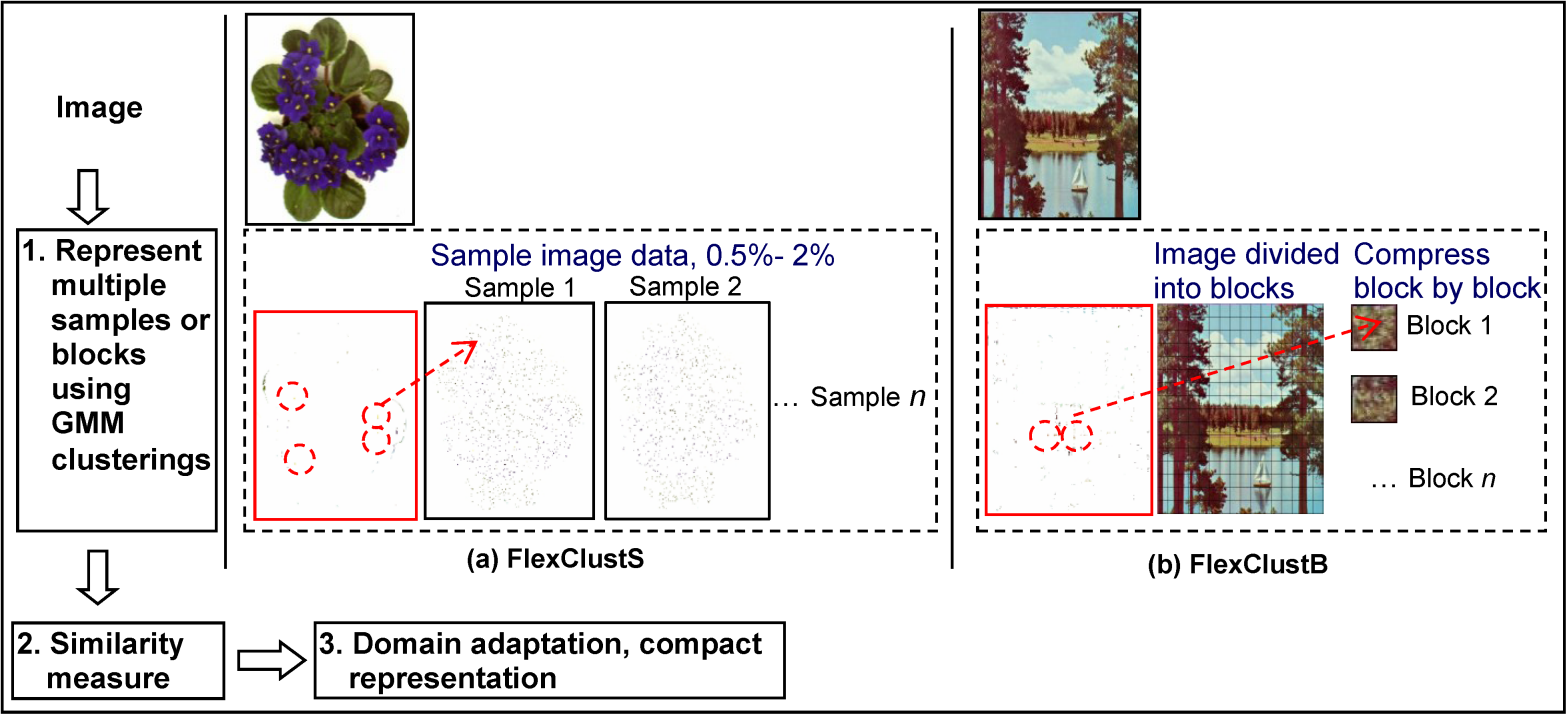
Overview of the proposed FlexClustS and FlexClustB algorithms.

To overcome the drawbacks of the algorithms for scaled down image data as described in Section 1, this paper proposes the FlexClustS and FlexClustB algorithms. The main idea of the proposed algorithms is to iterate over samples or blocks of the image data set. The three main modules in the algorithm are: (1) representation of the multiple samples or blocks of image using GMM guided by scan through and selection procedure, (2) calculation of the pairwise similarity measures of the descriptors of samples or blocks, and (3) domain adaptation to obtain a GMM compact representation of the entire image. The overview of the algorithms is given in Fig 1. The three modules of the algorithms will be described in the following sub-sections and the summary of the algorithms will be presented at the end of this section.

### 3.1 Represent multiple samples or blocks of image

In this paper, the scan through and selection procedure is proposed to improve the inclusion of the small details of the image. These pixels are isolated from the relatively small pixel clusters as follows:

1. Draw a block of image data *B* = {**x**_1_, **x**_2_, …, **x**_*nb*_} of *d*-dimension and size *n*_*b*_ = *p*_*s*_ *N*, where *p*_*s*_ is a small portion (say 1%) of the image data points, *N*. Perform *k*-means clustering on *B*. The number of pixel clusters is set as a priori by *k* = 0.01 *p*_*s*_ *N*. The aim of the *k*-means is to divide the *n*_*b*_ image data points into *k* pixel clusters in order to minimise an objective function given by
  $$D=\sum_{j=1}^{k}\sum_{i=1}^{n_{b}}{\left\|x_{i}^{\left(j\right)}-c_{j}\right\|}^{2},$$ (15)
  where || . ||^2^ is the Euclidean distance between **x**_*i*_^(*j*)^ and c_*j*_, and **x**_*i*_^(*j*)^ is the data point **x**_*i*_ from cluster *j*, and c_*j*_ is the centre of cluster *j*.
2. Consider there are *n*_*j*_ data points in cluster *j*. If the proportion of data points in cluster *j* (= *n*_*j*_/*N*) is less than a threshold, say *ε* = 0.01, consider them come from small pixel cluster, and let them be in set *Q*_*s*_.
3. Repeat steps (i) and (ii) until all the 100 blocks of the data points have been scanned through. Replicate set *Q*_*s*_ *q* times to over represent it to form set *Q*. This is to increase the chance to detect small pixel clusters in the later step. Adjust *q* according to the allowable memory for computation. In the case if the image has very fine structure with very small pixel clusters to be recovered, increase *p*_*s*_ and reduce *k* of *k*-means to increase the chance of capturing them in set *Q*_*s*_.

Consider now the image as being divided into multiple samples or blocks. The set *Q* is added to one of the samples or blocks of the image. Let *S*_1_ = {**x**_1_, **x**_2_, …, **x**_*n*1_} be the combination of *Q* and the first sample or block of the image data set. *S*_1_ is represented by the descriptors of GMM MLEs as follows:

1. Fit *S*_1_ into a *g*_1_-component Gaussian mixture model using the complete log-likelihood function in Eq (3). Repeat the step of fitting Gaussian mixture model for the remaining samples or blocks.
2. Let the MLEs of the parameter set for the *t-*th sample or block of image data be
  $${\hat{\Psi }}_{t}=({\hat{\theta }}_{t1},\ldots ,{\hat{\theta }}_{g_{t}},{\hat{\pi }}_{t1},\ldots ,{\hat{\pi }}_{tg_{t}}),\;\mathrm{where}\;{\hat{\theta }}_{tk}=({\hat{\mu }}_{tk},{\hat{\Sigma }}_{tk})$$
  is the vector of the MLE of mean and full covariance matrix, and ${\hat{\pi }}_{tk}$ is the MLE of mixture proportion, for the *k-*th cluster from the *t*-th portion, where *k* = 1, …, *g*_*t*_.
3. The MLEs of each individual cluster are estimated approximately from the decomposition of the mixture model components. Thus, for the *t*th portion, MLEs of the parameter set is decomposed into its mixture components
  $${\hat{\Psi }}_{tk}=({\hat{\mu }}_{tk},{\hat{\Sigma }}_{tk},n_{tk})$$
  for *k* = 1, …,*g*_*t*_, where *n*_*tk*_ = ${\hat{\pi }}_{tk}$ *n*_*t*_ is the *k*th cluster size.

Therefore, each of the image sample or block is now represented by the GMM MLE of pixel clusters given by the descriptors of

$${\hat{\Psi }}_{k}=({\hat{\mu }}_{k},{\hat{\Sigma }}_{k},n_{k}),$$ (16)

where *k* = 1, …, *g*_*t*_, and *g*_*t*_ is the number of clusters in the *t-*th sample or block.

### 3.2 Similarity measure: A modified Bayes factor

In this paper, a similarity measure using Bayesian approach based on model selection is proposed to distinguish between the homogeneous and heterogeneous clusters of pixels from different portions of image data. The proposed modified Bayes factor (MBF) works on the descriptors obtained from the previous step. For simplicity but without loss of generality, consider the descriptors from two portions of the image data. Let cluster *i* and cluster *j* be the pixel clusters from the first and second portion of the image data respectively. An assumption is made in developing the MBF: (i) if the two clusters *i* and *j* are similar, the clusters are merged for the later step, and (ii) if the two clusters *i* and *j* are dissimilar, the two clusters are maintained as they are for the further step. This notion actually implies the choice between two models each with the number of clusters *k* = 1 and *k* = 2 respectively, that is,

*M*1: *k* = 1,        if cluster *i* and cluster *j* are similar and can be merged,
*M*2: *k* = 2,        if cluster *i* and cluster *j* are dissimilar and cannot be merged,

see Section 3.3 for more details.

We choose the Bayesian approach for the above problem as it has advantages over the alternative frequentist hypothesis testing in the general context of model comparison; see [34] for details. The Bayesian application for pair wise models comparison and model selection is based on the Bayes factor [35–36]. Let **x** be the image data set for the pair of pixel clusters, the Bayes factor is given by the ratio of the posterior odds to its prior odds in favour of a model *M*_1_ over *M*_2_

$$B_{12}=p(M_{1}|x)/p(M_{2}|x)÷p(M_{1})/p(M_{2})=p(x|M_{1})/p(x|M_{2}).$$ (17)

The Bayes factor in Eq (17) is the likelihood ratio, and the densities, *p*(**x** | *M*_*i*_) for *i* = 1, 2, are obtained by integrating (not maximizing) over the parameter space given by

$$p(x|M_{i})=\int p(x|\theta_{i},M_{i})\pi (\theta_{i}|M_{i})d\theta_{i},$$ (18)

where *θ*_*i*_ is the parameter of *M*_*i*_, *π*(θ_i_ | *M*_*i*_) is the prior density of the parameter, and *p*(**x** | *M*_*i*_) is the probability density of **x** given *θ*_*i*_, or the likelihood function of *θ*_*i*_.

In practice, the marginal probability of the data, also termed as marginal likelihood or integrated likelihood, obtained from Eq (18) is often difficult to compute. [37] extended the Bayes factor for a standard comparison of nested hypotheses in the general linear model in the *p*-dimensional multivariate normal case with the following approximation:

$$-2\;{\log\;B}_{r,r+1}=\lambda -\left\{3/2+\log[\rho (n_{r,r+1})]\right\}\delta_{r,r+1},$$ (19)

where λ is the likelihood ratio test statistic, δ_*r*,*r*+1_ is the degree of freedom in the asymptotic chi-square distribution of λ, *n*_*r*,*r*+1_ is the number of data points in the merged cluster, and *ρ*(*n*_*r*,*r*+1_) is the rate of “shrinkage” of the prior covariance matrix which can be approximated by *n*_*r*,*r*+1_ when *n*_*r*,*r*+1_ is large. Unfortunately, the regularity conditions do not hold for λ to have its usual asymptotic null chi-square distribution with the degree of freedom δ_*r*,*r*+1_ in the clustering context. Based on a small scale simulation study of multivariate normal component densities with common covariance matrix for the number of clusters *k* = 1 versus *k* = 2, [38] suggested an approximation of 2δ_*r*,*r*+1_ to get around the problem.

In the proposed algorithms, the decision whether to select between the models with the number of clusters *k* = 1 and *k* = 2 for each of the pixel cluster pairs. Thus, we adapt a special case of Eq (19) when *r* = 1 with Wolfe’s approximation, and further assume that the merged pixel cluster size is large for image data clustering, to approximate the Bayes factor as follow

$$-2\;\log\;B_{r,r+1}=\lambda -\left\{3/2+\log[\rho (n_{r,r+1})]\right\}2\delta_{r,r+1}.$$ (20)

Let the maximum log-likelihood for the pair of pixel clusters involved in merger be log *L*_*i*_ and log *L*_*j*_ respectively, and the maximum log likelihood for the cluster resulting from the merger be log *L*_*m*_. Therefore, the term λ can be written as

$$\lambda =2(\log L_{i}+\log L_{j}-\log L_{m}).$$ (21)

From Section 3.1, the pixel clusters involved in the merger are described by their MLEs decomposed from Gaussian mixture models. Therefore, the merged pixel cluster will be described by the weighted MLEs of the pair of pixel clusters (see Section 3.3). The maximum log-likelihood functions of the paired and the merged clusters are of the same form which is given by

$$\begin{array}{l} \log L(\hat{\mu },\hat{\Sigma })=-(np/2)\log(2\pi )-(n/2)\log\left|\hat{\Sigma }\right|-1/2\sum_{i=1}^{n}{\left(x_{i}-\hat{\mu }\right)}^{T}\Sigma^{-1}(x_{i}-\hat{\mu })\\ \;=-(np/2)\log(2\pi )-(n/2)\log\left|\hat{\Sigma }\right|-(1/2)trace(W{\hat{\Sigma }}^{-1}) \end{array}$$

where

$$W=\sum_{i=1}^{n}{\left(x_{i}-\hat{\mu }\right)}^{T}(x_{i}-\hat{\mu })=\hat{\Sigma }.$$

Thus, the concentrated log-likelihood is

$$\log L(\hat{\mu },\hat{\Sigma })=-(np/2)\log(2\pi )-(n/2)\log|\hat{\Sigma }|-np/2.$$ (22)

Substituting Eq (22) for the paired and the merged clusters, and Eq (21) in Eq (20), we get the proposed modified Bayes factor (MBF) as

$$-2\;{\mathrm{logB}}_{r,r+1}=-n_{1i}\log\left|{\hat{\Sigma }}_{1i}\right|-n_{2j}\log\left|{\hat{\Sigma }}_{2j}\right|+n_{m}\log\left|{\hat{\Sigma }}_{m}\right|-2\left(d+d(d+1)/2\right)\left\{3/2+\log(n_{m})\right\}.$$ (23)

The MBF suggests the choice of models based on the change in log-likelihood as a result of merging the pair of pixel clusters together. From Eq (23), it can be seen that the smaller the generalized variance the larger is the log-likelihood. Thus, if MBF is positive, the merged cluster gives bigger generalized variance and smaller log-likelihood (more negative) than the pair of pixel clusters, and this suggests that the pair of pixel clusters should not be merged, or in other words, they are dissimilar. On the other hand, if the MBF is negative, the merged cluster gives smaller generalized variance and larger log-likelihood (less negative), and the pair of pixel clusters should be merged, which implies that the clusters are similar.

The main advantage of the proposed MBF similarity measure is not only to provide information for the compact representation for the entire image by merging similar clusters to produces higher maximum log-likelihood, but also information for domain adaptation.

### 3.3 Domain adaptation and compact representation

A mixture model distribution is proposed to aggregate the sets of local image descriptors in the format of GMM MLEs into a compact representation of the entire image. As the different image samples or blocks may have different numbers of descriptors and some descriptors may consist of novel local features, domain adaptation will be performed.

Consider the GMM representation of *S*_1_ in Section 3.1 as source domain, the descriptors from the other samples or blocks are in the target domain. Let

$$({\hat{\mu }}_{1},{\hat{\Sigma }}_{1},n_{1a})\in {\hat{\Psi }}_{1k}=({\hat{\mu }}_{1k},{\hat{\Sigma }}_{1k},n_{1k}),\;k=1,\ldots ,g_{1},$$

and

$$({\hat{\mu }}_{2},{\hat{\Sigma }}_{2},n_{2a})\in {\hat{\Psi }}_{2k}=({\hat{\mu }}_{2k},{\hat{\Sigma }}_{2k},n_{2k}),\;k=1,\ldots ,g_{2},$$

be the decomposed MLEs of the pair of pixel clusters from the source and target domain respectively, and

$$\left({\hat{\mu }}_{m},{\hat{\Sigma }}_{m},n_{ma}\right)$$

be the MLE of sufficient statistics (*μ*_*m*_, *Σ*_*m*_), where *n*_*ma*_ is the cluster size of the merged cluster. If MBF suggests that the two descriptors are similar and can be merged, the parameters of the GMM model trained from *S*_*1*_ are updated using weighted MLEs as follows:

1. The MLEs for the merged cluster are estimated from
  $$\begin{array}{l} n_{ma}=n_{1a}+n_{2a},\\ {\hat{\mu }}_{m}=\sum_{j=1}^{2}n_{ja}{\hat{\mu }}_{j}/n_{ma},\\ {\hat{\Sigma }}_{m}=\left(\sum_{j=1}^{2}n_{ja}({\hat{\mu }}_{j}-{\hat{\mu }}_{m})({\hat{\mu }}_{j}-{\hat{\mu }}_{m})+\sum_{j=1}^{2}n_{ja}{\hat{\Sigma }}_{j}\right)/n_{ma}. \end{array}$$ (24)
2. The mixture proportions of the trained model become
  $${\hat{\pi }}_{m}{}^{*}=\left(n_{1a}+n_{2a}\right)/\left(n_{1}+n_{2a}\right),$$ (25)
  for the component involved in merging; and
  $$\pi_{k}{}^{*}=n_{1k}/\left(n_{1}+n_{2a}\right),$$ (26)
  for the other components.

The GMM model is now updated to

$$\begin{array}{l} f(x_{i}|\Psi )=\sum_{k=1}^{g_{1}-1}\pi_{k}{}^{*}\varphi_{k}(x_{i}|\mu_{k},\Sigma_{k})+\pi_{m}{}^{*}\varphi (x_{i}|\mu_{m},\Sigma_{m})\\ \;=\sum_{k=1}^{g_{1}}\pi_{k}{}^{*}\varphi_{k}(x_{i}|\mu_{k},\Sigma_{k}),\;i=1,2,\ldots n_{1}+n_{2a}. \end{array}$$ (27)

On the other hand, if the MBF suggests that the two descriptors are dissimilar, domain adaptation will be performed by adding a new mixture component. The mixture proportions of the model are updated as follows:

$$\pi_{2a}{}^{*}=n_{2a}/(n_{1}+n_{2a}),$$ (28)

for the new added cluster;

$$\pi_{k}{}^{*}=n_{1k}/\left(n_{1}+n_{2a}\right),$$ (29)

for the other existing components.

The GMM model is now given by

$$\begin{array}{l} f(x_{i}|\Psi )=\sum_{k=1}^{g_{1}}\pi_{k}{}^{*}\varphi_{k}(x_{i}|\mu_{k},\Sigma_{k})+\pi_{2a}{}^{*}\varphi (x_{i}|\mu_{2},\Sigma_{2})\\ \;=\sum_{k=1}^{g_{1}+1}\pi_{k}{}^{*}\varphi_{k}(x_{i}|\mu_{k},\Sigma_{k})\;i=1,2,\ldots n_{1}+n_{2a}, \end{array}$$ (30)

where

$$\pi_{\left(g_{1}+1\right)}^{*}=\pi_{2a}{}^{*}.$$

The compact representation of the entire image is obtained through incremental model updates. In each of the iteration in the model update, only the GMM MLEs are used. The domain adaptation is performed over two sets of MLEs instead of revisiting the pixel data points. Hence, the proposed FlexClustS and FlexClustB clustering algorithms are scalable to very large image data sets. In the reconstruction of image using the GMM compact representation, the mixture component without any assignment will be considered as spurious component and therefore be removed as it has almost no negative impact on the model quality [39].

The FlexClustS and FlexClustB algorithms are summarized in Algorithms 1 and 2 respectively.

Algorithm 1.FlexClustS.

Stage 1:

    Isolate the small pixel clusters using Eq (15).

Stage 2:

    Divide image into samples. Add isolated pixels to one of these samples.

    Represent each sample using Eq (3), and using descriptor given by Eq (16).

Stage 3:

    Calculate the similarity measure between descriptors obtained from Stage 1 using Eq (23).

    Aggregate the sets of local image descriptors based on the similarity measures.

    If descriptors are similar, update GMM model using Eqs (24–26). The representation in GMM is given by Eq (27).

    If descriptors are dissimilar, perform domain adaptation, update GMM model using Eqs (28) and (29). The representation in GMM is given by Eq (30).

Algorithm 2. FlexClustB.

Stage 1:

    Isolate the small pixel clusters using Eq (15).

Stage 2:

    Divide image into blocks. Add isolated pixels to one of these blocks.

    Represent each block using Eq (3), and using descriptor given by Eq (16).

Stage 3:

    Calculate the similarity measure between descriptors obtained from Stage 1 using Eq (23).

    Aggregate the sets of local image descriptors based on the similarity measures.

    If descriptors are similar, update GMM model using Eqs (24–26). The representation in GMM is given by Eq (27).

    If descriptors are dissimilar, perform domain adaptation, update GMM model using Eqs (28) and (29). The representation in GMM is given by Eq (30).

## 4 Experimental evaluation

### 4.1 Algorithms for comparison

The performance of the proposed FlexClustS and FlexClustB is compared to two existing mixture model algorithms: Strategy III [1] (See Section 2.3) and sufficient EM [25] (See Section 2.2) respectively. Strategy III and sufficient EM are chosen to represent respectively the sampling based and blocks based methods of processing scaled down image data mentioned in Section 1. Strategy III applies a mixture model clustering to a sample of the full data, and then extends five tentative best models from the sample via EM to the full data in more iteration to eventually select the best model from the tentative best models. Sufficient EM is a variant of EM used in parameter estimation for mixture model clustering of multiple sets of sufficient statistics (i.e. means and covariance, and the number of data points). Each set of sufficient statistics characterizes a dense region of data points that is obtained by *k*-means clustering.

### 4.2 Data

Three set of simulation data with known cluster label and five sets of image data i.e. *St Paulia*, *cytology*, *Lena*, s*ailboat* and *San Diego* are used to evaluate the performance of the proposed algorithms.

The first set of simulated data consists of 15,000 data points generated from a seven-component two-dimensional Gaussian mixture distribution. Special attention is paid to the relatively small nested Cluster-6. The parameters for the data set are as follows:

Cluster-1:        (*μ*1,*Σ*1,*n*1) = ((−10,38), (1.5,−1,−1,20), 2000),
Cluster-2:        (*μ*2,*Σ*2,*n*2) = ((−6,35), (20,0,0,1), 4000),
Cluster-3:        (*μ*3,*Σ*3,*n*3) = ((7,30), (6,0.5,0.5,3), 3500),
Cluster-4:        (*μ*4,*Σ*4,*n*4) = ((30,60), (3,0,0,33), 1000),
Cluster-5:        (*μ*5,*Σ*5,*n*5) = ((−25,35), (8,−0.1,−0.1,1), 2000),
Cluster-6:        (*μ*6,*Σ*6,*n*6) = ((−29,34), (0.5,0,0,0.5), 1000),
Cluster-7:        (*μ*7,*Σ*7,*n*7) = ((−50,50), (0.5,3,3,20), 1500).

The second and third sets of simulated data are generated using the population parameters of the *wine* and *iris* data set from UCI machine learning repository [40] that are fitted to the three-component VVI model and three-component VEV model [21, 41] respectively (available at http://archive.ics.uci.edu/ml/datasets.html). The generated *wine* and *iris* data sets are of sizes 20,000 and 10,000 respectively. The *wine* data set is concerned with the chemical quantities of 13 constituents found in each of the three types of wines grown in the same region in Italy. It has “well behaved” class structures. The *iris* data set contains 3 classes (Versicolor, Virginica, and Setosa) of iris plant based on the measurement of four features i.e. sepal length and width, petal length and width. Two of the three classes in the *iris* data are overlapping. These three sets of simulated data are calibrated using MixSim [42]. The calibration of data set is based on the criteria of average pairwise overlap and maximum pairwise overlap [43]. The calibration results are shown in Table 1.

**Table 1:** Calibration results of simulated data sets using MixSim.

Simulated data	Average pairwise overlap	Maximum pairwise overlap	Degree of overlap for the clusters
Data Set 1	0.0199	0.2481	High
Data Set 2	0.0013	0.0024	Low
Data Set 3	0.0165	0.0495	Moderate

Based on [43], the interpretation for degree of component overlap from pairwise overlaps value is: well separated (below 0.05), moderate separated (between around 0.05 and 0.1), and poorly separated (above 0.15). Therefore, the clusters of the first simulated data set have the highest degree of overlap and followed by the third data set and second data set.

Five sets of RGB image data *St Paulia*, *cytology*, *Lena*, s*ailboat* and *San Diego* are considered for application. *St Paulia* (304 x 238 pixels) is a flower image which has been used in [1]. Identifying the small yellow flowers is of particular interest. *Cytology* (248 x 150 pixels) is obtained from the Internet (https://commons.wikimedia.org/wiki/File:Canine_transmissible_venereal_tumor_cytology.JPG, owned by Joel Mills). Identifying the details of the cell structure is the focus. The image of *St Paulia* and *Cytology* are contributed in supplementary information files (S1 Fig and S2 Fig). *Lena*, s*ailboat* and *San Diego* (512 x 512 pixels) are three well-known benchmark images selected from the Berkeley Segmentation Data Set (BSDS) [44] to represent portrait, landscape and satellite images (available at https://www.eecs.berkeley.edu/Research/Projects/CS/vision/bsds/).

### 4.3 Evaluation criteria

The performance of FlexClust is assessed according to three main aspects of (i) how well the features in partial data are captured, (ii) how well the descriptors from different partial data are classified, and (iii) how well the recovery of small but important clusters which are incorporated through domain adaptation into the GMM compact representation of the entire data set.

With the known cluster label for each data point of the simulated data, the performance of the capture of cluster features and classification of the descriptors are evaluated through the partitioning error and labelling error. The partitioning error is measured by Adjusted Rand Index (ARI) [45]. Given a set of *n* objects with two partitions *U* and *V*, the ARI is a chance-corrected measure of agreement about the number of pairs of objects that belong to the same group and different groups between the two partitions as summarized below:

Let a = number of pairs of objects in the same group in Partition *U* and also Partition *V*,
b = number of pairs of objects in the same group in Partition *U* but in the different groups in Partition *V*,
c = number of pairs of objects in the different groups in Partition *U* but in the same group in Partition *V*,
d = number of pairs of objects in the different groups in Partition *U* and also the different groups in Partition *V*.

The ARI is defined as

$$\mathrm{ARI}=\frac{\left(\begin{array}{c} n\\ 2 \end{array}\right)(a+d)-[(a+b)(a+c)+(c+d)(b+d)]}{{\left(\begin{array}{c} n\\ 2 \end{array}\right)}^{2}-[(a+b)(a+c)+(c+d)(b+d)]}$$

ARI is equal to one for perfect agreement, and takes negative value if the agreement is lower than what is expected by chance. The labelling error is measured by misclassification error which is the proportion of data points that is clustered into the wrong group. The aspect of incorporation of novel clusters through domain adaptation is assessed by model fit and the number of clusters in the final GMM compact representation of the entire data set. The value of log-likelihood is used to assess the model fit.

For image data, the true class label of every pixel is normally unavailable. A more objective performance measure on different clustering algorithms for image processing should involve assessment between the reproduced images and the reference (or ground true or original) image quality. The simplest and most widely used image quality metric is mean square error (MSE), where the intensity differences of the reproduced image and the reference image pixels are squares and then averaged. However, [46] showed that the images with different degree of distortions altered from the same original image with drastically different perceptual quality based on human visual are having nearly identical MSE. [46] developed structural similarity index (SSI) for measuring the similarity between two aligned image signals. The SSI is a quantitative measurement of the quality of an image provided a reference image is regarded as of perfect quality. It is a combination of three components namely luminance, contrast and structural components. The comparison of images is based on the estimates of intensity mean shift for luminance, change of intensity standard deviation for contrast, and change of normalized signal intensity for structure or the collective remaining errors. The application of SSI for image processing evaluation has been rapidly increasing and become a widely accepted image quality metrics. In this section, the qualities of the images processed by FlexClust, Strategy III and sufficient EM are assessed by comparing to the ground true image, and the SSIs are computed. When SSI is equal to one, it indicates there is no loss of information in the reconstructed image, and the nearer SSI is to 1, the better the image quality. All the SSIs in this study are implemented in ssim.m [47]. The images are also assessed visually based on qualitative evaluation as human visual is efficient to detect if there is loss of image detail [48].

### 4.4 Experiment setting

For the comparison of the sampling based algorithms, the initial sample sizes in Strategy III are set equal to the sample sizes of FlexClustS. Two different sample sizes with 10 experiments each are considered so that the conclusions do not depend on the sample size and particular sample drawn. With consideration of reasonable computation time for the EM algorithm, the sample sizes selected for images with 23712 pixels to 262144 pixels range from 500 to 2500 pixels [1]. At the same time, this paper also intends to evaluate the effectiveness of the algorithms by using rather small proportion of the image as the sample size. Therefore, the sample sizes considered are 1% and 2% of the image data, which are 723 and 1447 pixels for *St Paulia*, 372 and 744 pixels for *cytology* respectively. For larger image size, the sample sizes considered are 0.5% and 1% for *Lena*, *sailboat* and *San Diego*, which are 1310 pixels and 2621 pixels.

In the evaluation of the block based algorithms, each image is divided into two different numbers of blocks and the sufficient statistics of the pixel clusters are obtained from each block. The block sizes are 8x2 and 16x2 for *St Paulia*, 5x4 and 10x8 for *cytology*, and are 8x8 and 16x16 for *Lena*, *sailboat* and *San Diego*. The total number of sets of resulting sufficient statistics is set about the same as the portion size in FlexClustB. However, if the set of sufficient statistics consists of local dense region with only one data point, the number of sets of sufficient statistics has to be reduced so that the covariance exists.

All the clustering algorithms consider 2 to 10 clusters for the simulated data and 3 to 15 clusters for the image data. The clustering of all the experiments in FlexClustS, FlexClustB and Strategy III are performed using MCLUST [21, 41] which considers ten parameterizations of the cluster covariance matrices and uses solution obtained from hierarchical clustering for initialization of the EM algorithm. The selection of MCLUST is based on its comprehensive strategy for clustering, classification and density estimation for Gaussian mixture model, which are in line with the objectives of this paper. See [49] for more details on the capability comparison of R packages for Gaussian mixture modelling. For FlexClustS and Strategy III only the four most elaborate models i.e. EEE, EEV, VEV and VVV [1] in MCLUST are considered. The maximum number of iterations for all the three algorithms is set as 100. For the simulated data, the sufficient statistics for sufficient EM are obtained from summarizing the dense regions of the whole data set.

### 4.5 Result on simulation

Results of the simulation study are shown in Table 2. For the sampling based algorithms, FlexClustS outperforms Strategy III in terms of the agreement of partition, agreement of class label, and model fit in the well separated and moderated separated components of Data Sets 2 and 3 respectively regardless of the sample size. FlexClustS also performs better than Strategy III in the poorly separated components of Data Set 1 in terms of agreement of partition and model fit when different sample sizes are used. However, the labelling error for this poorly separated components data set is influenced by the sample size. The labelling error of FlexClustS is slightly higher than Strategy III when the sample size is 500 but much lower than Strategy III when the sample size increases to 1000. For the partition based algorithms, FlexClustB outperforms sufficient EM in terms of the agreement of partition, agreement of class label, and model fit for data sets with different degrees of component overlap.

**Table 2:** Average performance measures of the 3 simulated data sets.

Method	Data Set 1				Data Set 2				Data Set 3			
	ARI	Error	Loglik	*cK*	ARI	Error	Loglik	*cK*	ARI	Error	Loglik	c*K*
	*n* = 500				*n* = 250				*n* = 250			
FlexClustS	**0.8389**	0.1102	**-96113**	**100%**	**0.9956**	**0.0014**	**-371642**	**100%**	**0.9294**	**0.0336**	**-14577**	**100%**
Strategy III	0.8315	**0.1014**	-97744	0%	0.9827	0.0116	-391753	20%	0.9254	0.0404	-17638	50%
	*n* = 1000				*n* = 500				*n* = 500			
FlexClustS	**0.8446**	**0.0996**	**-96116**	**50%**	**0.9956**	**0.0014**	**-371642**	**100%**	**0.9478**	**0.0181**	**-12805**	**100%**
Strategy III	0.7905	0.1273	-100439	0%	0.9387	0.0637	-406970	0%	0.9004	0.0654	-17313	40%
	*n* = 500 or *np* = 500				*n* = 250 or *np* = 250				*n* = 250 or *np* = 250			
FlexClustB	**0.8728**	**0.0587**	**-90706**	**100%**	**0.9956**	**0.0014**	**-371650**	**100%**	**0.9464**	**0.0186**	**-12825**	**100%**
Sufficient EM	0.8600	0.0786	-95170	50%	-0.0072	0.6048	-440091	10%	0.8916	0.0598	-14670	**100%**
	*n* = 1000 or *np* = 800				*n* = 500 or *np* = 500				*n* = 500 or *np* = 500			
FlexClustB	**0.8731**	**0.0589**	**-90683**	**100%**	**0.9956**	**0.0014**	**-371652**	**100%**	**0.9459**	**0.0188**	**-12831**	**100%**
Sufficient EM	0.8538	0.0792	-93862	40%	0.0319	0.5655	-449133	20%	0.9437	0.0196	-12807	**100%**

Note: ARI is the adjusted Rand index, Error is the misclassification error, Loglik is the log-likelihood, c*K* is the percentage of getting the correct number of clusters, *n* is the sample size, *np* is the number of prototypes.

**Fig 2:**
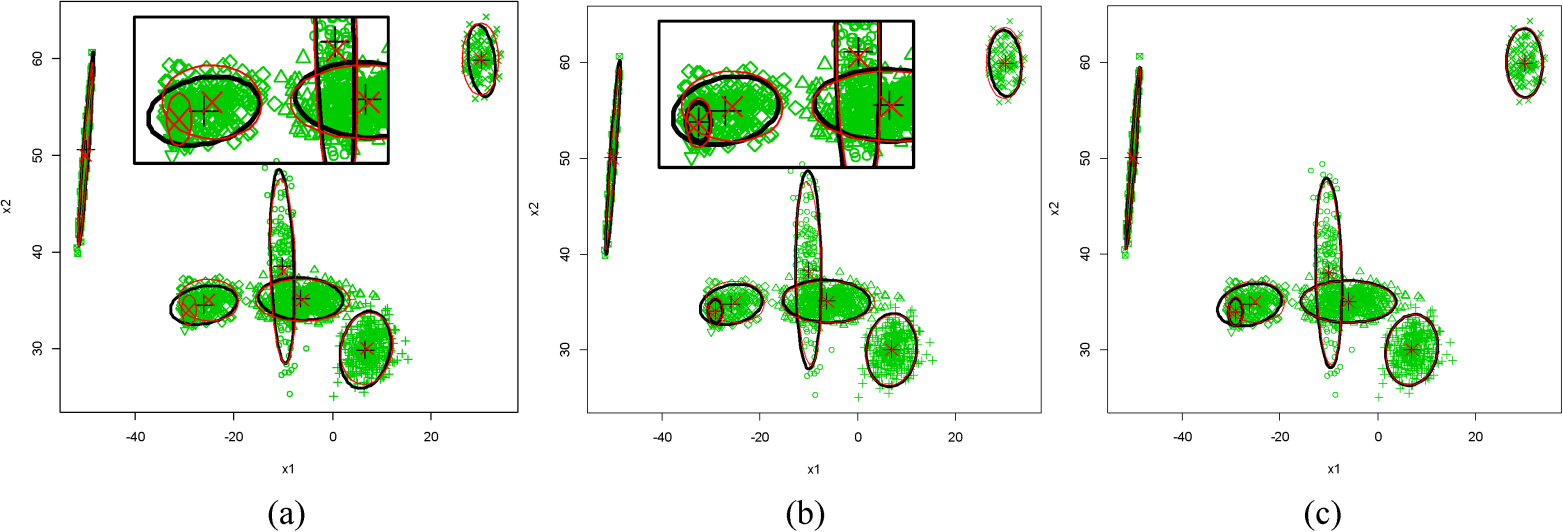
True parameter of means and covariances (‘x’ and red line) compared to the FlexClustS model (‘+’ and black line). Comparison after processing: a) 1 sample, b) 3 samples, and c) whole data set, in one of the experiments of FlexClustS [500]. (Note: ‘x’ and ‘+’ represent means, and covariances are visualized by 90% normal tolerance ellipsoids).

**Fig 3:**
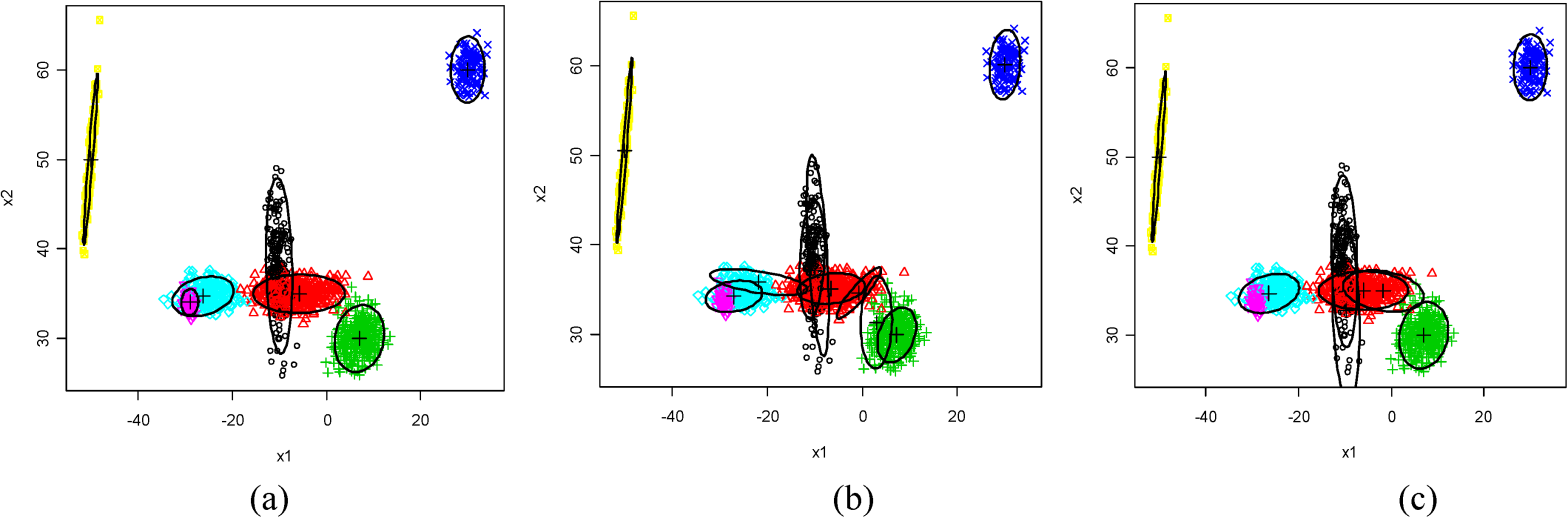
True clusters structure (scatterplot of 10% of the total data points), and the examples of cluster structure obtained by three algorithms. (a) FlexClustS–identify the correct 7 clusters, (b) Strategy III—miss out cluster 6 but identify superfluous clusters, (c) sufficient EM—miss out cluster 6 but identify superfluous clusters at cluster 1 and 2.

Based on the c*K*, FlexClustS performs more consistently and accurately in determining the correct number of clusters than other algorithms for different degrees of component overlap and dimensions of data set. FlexClustS only does not 100% times correctly identify the number of clusters in Data Set 1 with sample size 1000, but it is still better than other algorithms.The model update of FlexClustS in Data Set 1 is used to illustrate how MBF makes FlexClustS outperforms other algorithms in recovering the novel local feature that has not being identified in the early portion of data, and how the proposed domain adaptation in model update helps to estimate the model parameters closer to the actual value and results in higher log likelihood values. In Fig 2(A), the MLE of means of the initial sample with the sizes of 500 shows apparently that Cluster-6 is not found at this stage, and the MLE of means are further from the actual means. In the third sample, Cluster-6 is identified as depicted in Fig 2(B), and the MBF suggests it is a new cluster. From Fig 2(B) to 2(C), no new cluster is found, and the MLE of means are closer to the actual means. Strategy III and sufficient EM tend to overestimate the number of clusters and identify superfluous components or even identical clusters. Fig 3 shows the examples of cluster structure obtained from the three algorithms.

The results show that the effects of initial sample and sample size are very minimal for all the algorithms. However, like other sampling based algorithms, sample size does affect the performance of FlexClustS in determining the correct number of clusters and the agreement of class label when the components are poorly separated. The complexity in terms of number of clusters of the final model obtained by FlexClustS is observed to increase with the sample size. More clusters are used to describe the sample especially at the overlapping area between the elongated Cluster 1 and Cluster 2 when the portion size increases.

**Fig 4:**
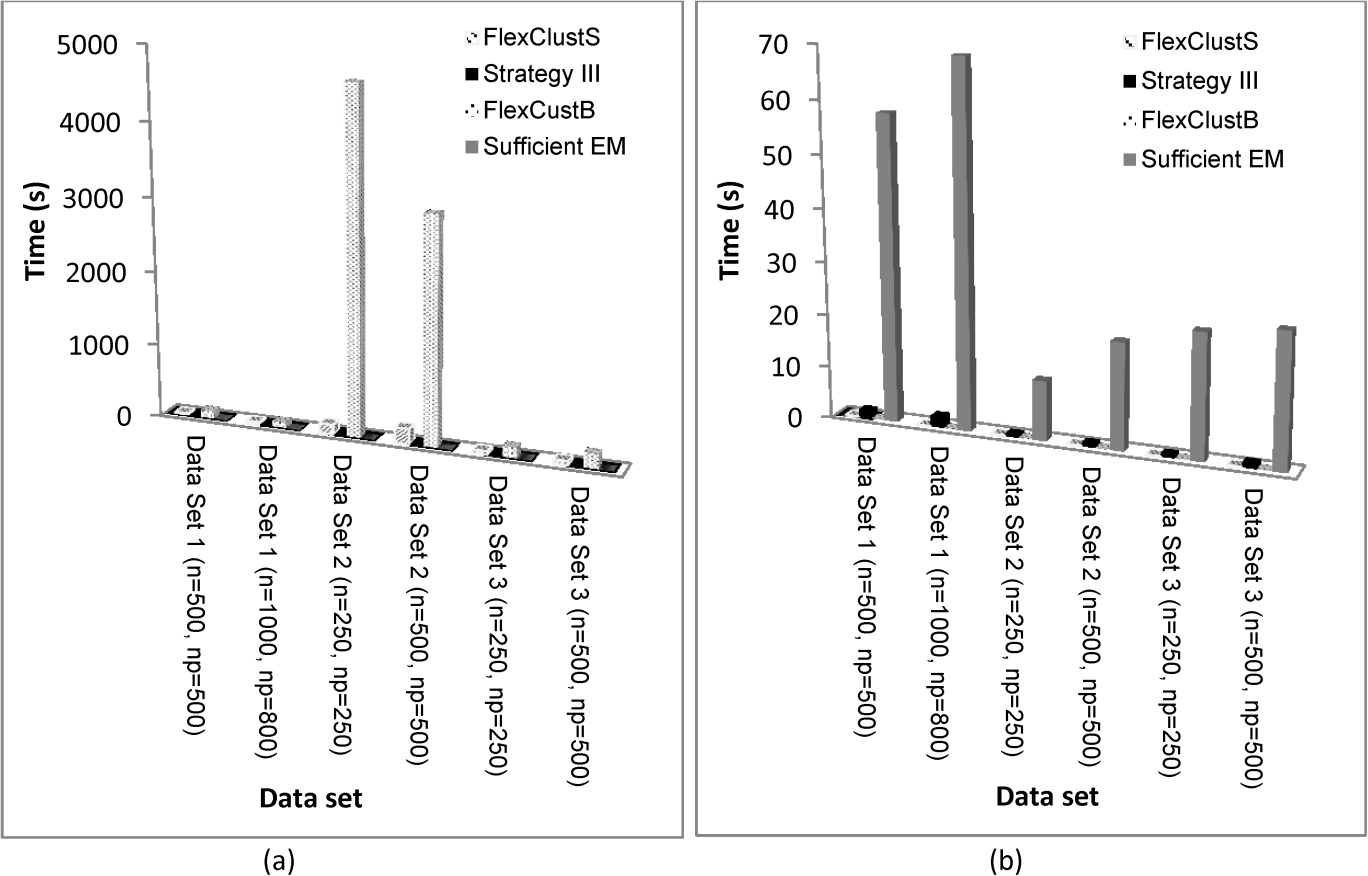
Comparison of computation time of the four algorithms for the simulated data. Computation Time in the stages of: (a) scaled down data clustering, (b) extend to all data clustering. (See Table 1 for the values of *n* and *np*).

**Fig 5:**
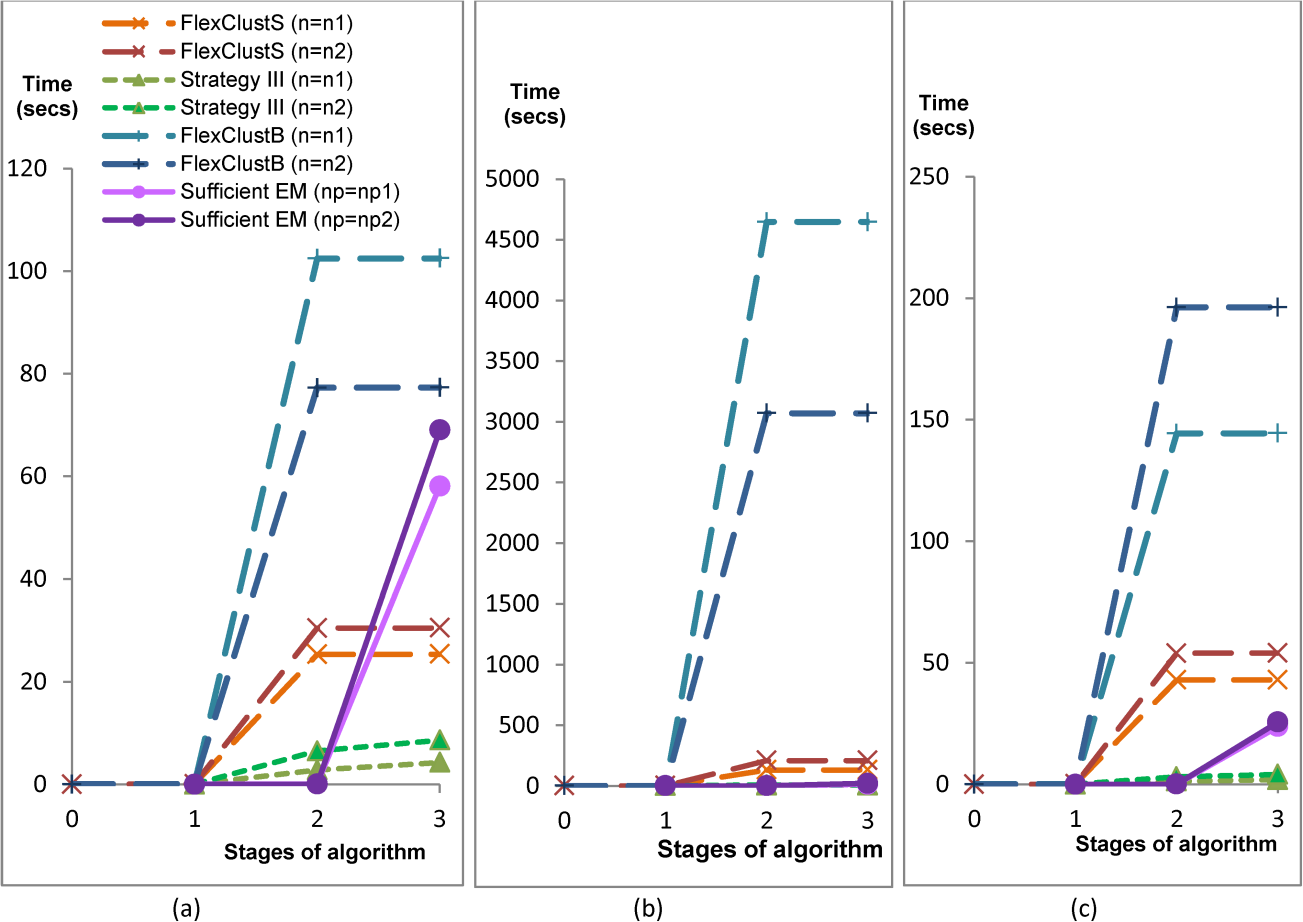
Accumulated stage by stage computational time of FlexClustS, Strategy III, FlexClustB and sufficient EM. (a) Simulated data set 1, (b) Simulated data set 2, (c) Simulated data set 3. (Note: FlexClustS and FlexClustB consists of 3 stages: (1) scan through and selection, (2) multiple samples clustering, (3) measure similarity and perform domain adaptation to classify all the descriptors. Strategy III consists of 2 stages: (1) one sample clustering, (2) EM to whole data set. Sufficient EM consists of 2 stages: (1) cluster whole data set by *k*-means, (2) EM to sufficient statistics obtained from stage (1)).

In terms of computational time as shown in Figs 4 and 5, FlexClustS is slower than Strategy III, and FlexClustB is slower than sufficient EM in the 2-dimensional Data Set 1. However, FlexClustS runs faster than sufficient EM in this data set. For higher dimensional data sets, FlexClustS and FlexClustB take longer time than the competitor algorithms. Fig 4 shows that FlexClustS and FlexClustB take more time to cluster the portions of data into multiple GMM, but works very fast when aggregating all the descriptors of MLEs.

### 4.6 Results on images

#### 4.6.1 Evaluation of image quality

**Fig 6:**
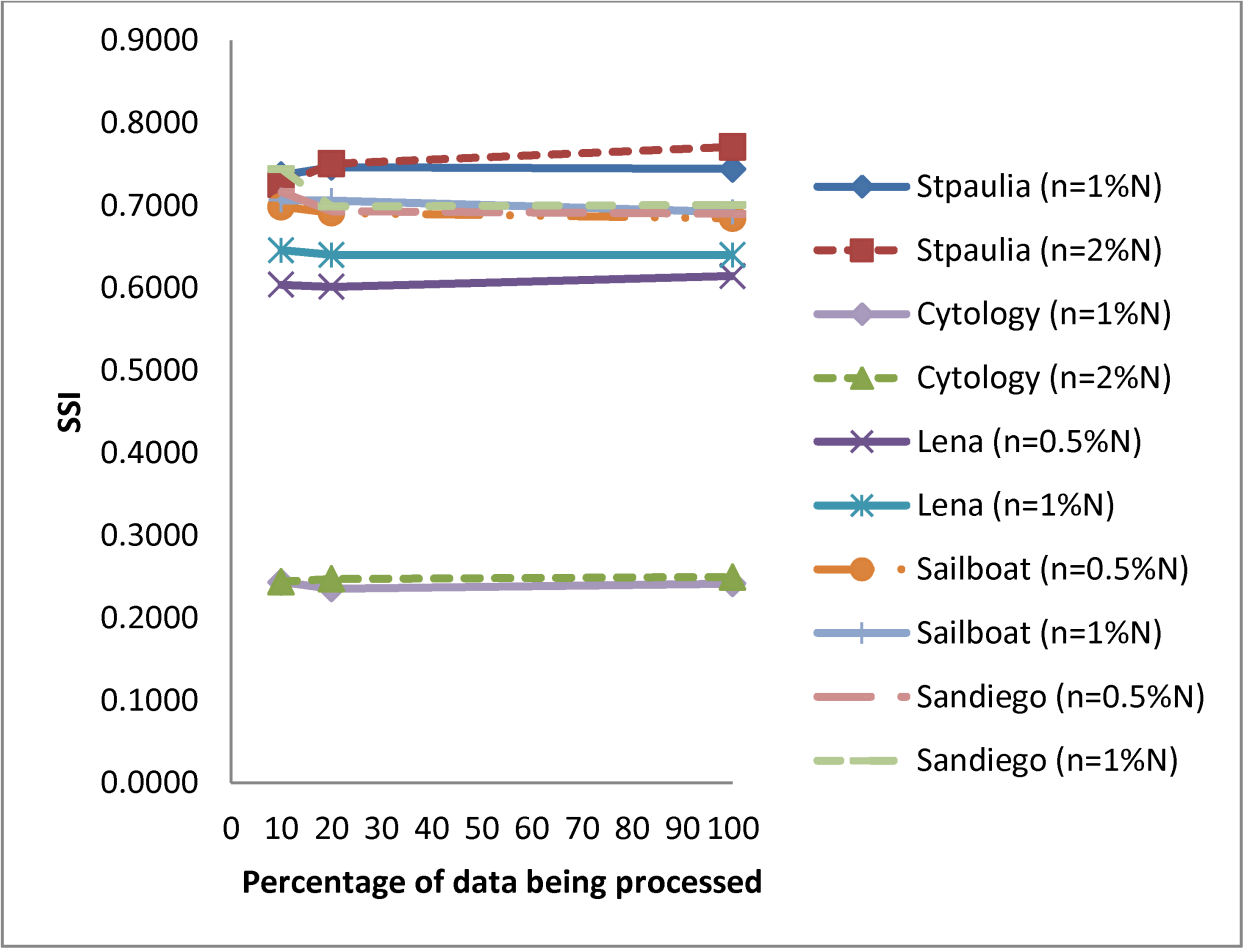
Structural similarity indices (SSI) of images being processed from 10%*N* to 100%*N* using FlexClustS.

Results of images processed through sampling are shown in Table 3. FlexClustS reproduces better quality image based on structural similarity index for *St Paulia*, *cytology* and *sailboat* regardless of the sample size used. FlexClustS with larger sample sizes reproduces all images with slightly higher SSIs than the smaller sample size. However, the same result is not observed in Strategy III. With larger sample sizes, Strategy III reproduces slightly higher SSI for *cytology* and *sailboat*, but not *St Paulia*, *Lena* and *San Diego*. Although the sample size selection influences the final result, its effect is very minimal. Furthermore, when the SSIs are compared between FlexClustS and Strategy III on the same image across different sample sizes, the results are consistent. It is interesting to note that even when FlexClustS processes only 10% of the image data, the SSI of these images are better than those obtained by Strategy III. Fig 6 shows that the SSI does not change much when FlexClustS processes from 10% to 100% of the image data. Some SSIs improve as more percentage of the image data is processed but some decline. For *San Diego*, the SSI of FlexClustS [*n* = 1%*N*] after processing 10% of the image data is higher than Strategy III, but lower after processing the whole image data.

**Table 3:** Structural similarity indices (SSI) for images processed through sampling.

Method	Process data until	St Paulia		Cytology		Lena		Sailboat		San Diego	
		(*n* = 1%*N*)	(*n* = 2%*N*)	(*n* = 1%*N*)	(*n* = 2%*N*)	(*n* = 0.5%*N*)	(*n* = 1%*N*)	(*n* = 0.5%*N*)	(*n* = 1%*N*)	(*n* = 0.5%*N*)	(*n* = 1%*N*)
FlexclustS	10%N	0.7365	0.7254	0.2432	0.2436	0.6036	0.6455	0.6983	0.7063	0.7161	0.7441
	20%N	0.7465	0.7504	0.2352	0.2469	0.6012	0.6400	0.6909	0.7059	0.6928	0.6988
	100%N	**0.7442**	**0.7707**	**0.2413**	**0.2492**	0.6143	0.6400	**0.6844**	**0.6920**	0.6897	0.7006
Strategy III	100%N	0.7285	0.7160	0.2337	0.2388	**0.6681**	**0.6671**	0.6827	0.6897	**0.7287**	**0.7184**

Note: *n* is the sample size, *N* is the image data size.

**Fig 7:**
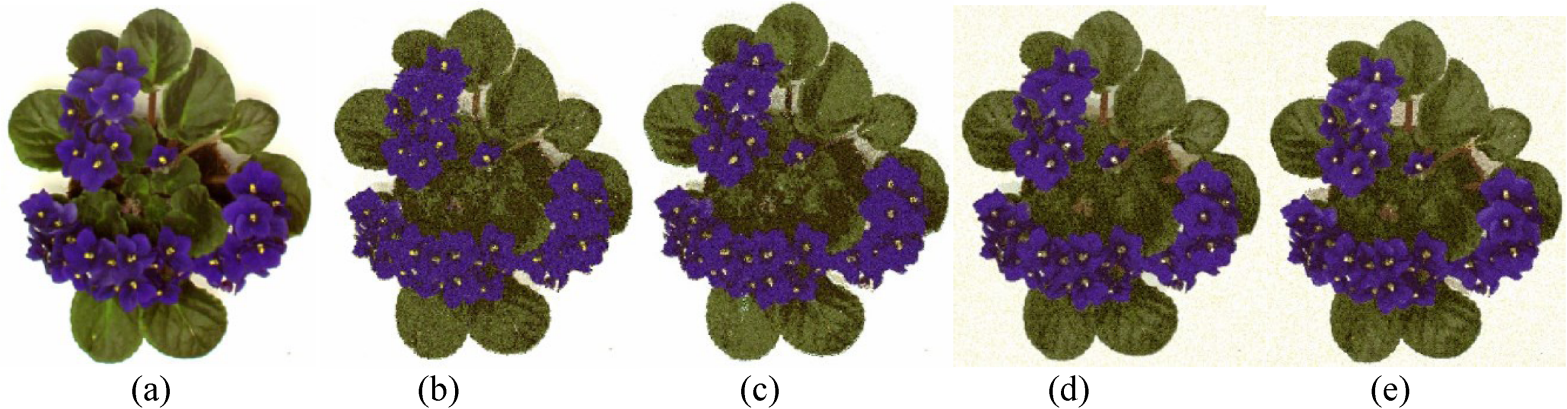
Comparison of *St Paulia* image. (a) Ground true (SSI = 1). Example of images obtained by: (b) FlexClustS [*n* = 1%*N*] (SSI = 0.7408), (c) FlexClustS [*n* = 2%*N*] (SSI = 0.7697), (d) Strategy III [*n* = 1%*N*] (SSI = 0.6970), (e) Strategy III [*n* = 2%*N*] (SSI = 0.7086).

**Fig 8:**
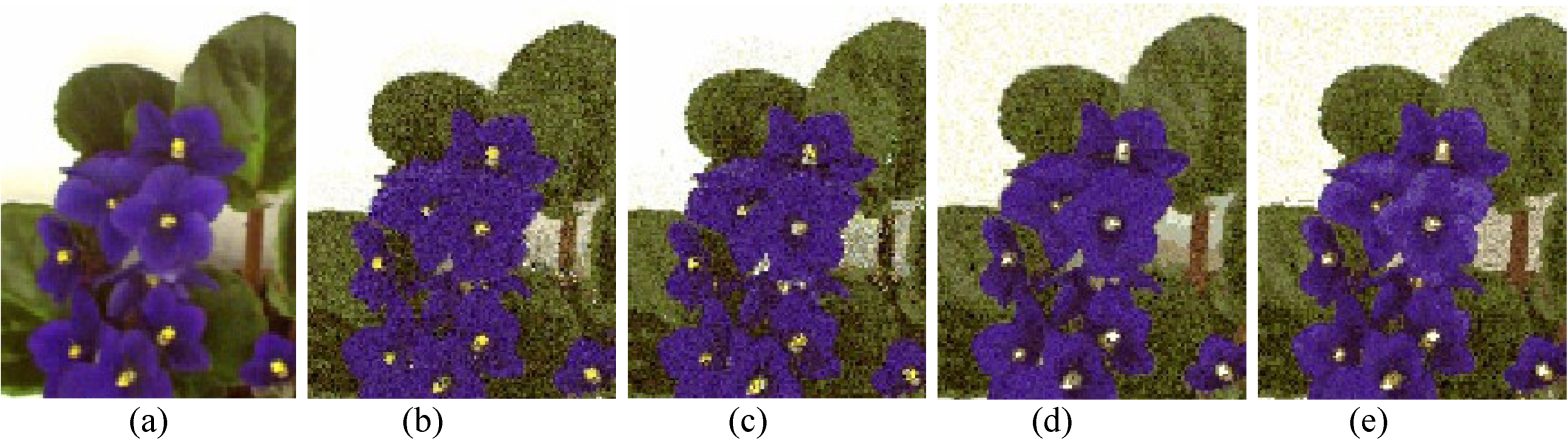
Close-up performance comparison of *St Paulia* image. (a) ground truth (SSI = 1), (b) FlexClustS [*n* = 1%*N*] (SSI = 0.7408), (c) FlexClustS [*n* = 2%*N*] (SSI = 0.7697), (d) Strategy III [*n* = 1%*N*] (SSI = 0.6970), and (e) Strategy III [*n* = 2%*N*] (SSI = 0.7086).

**Fig 9:**
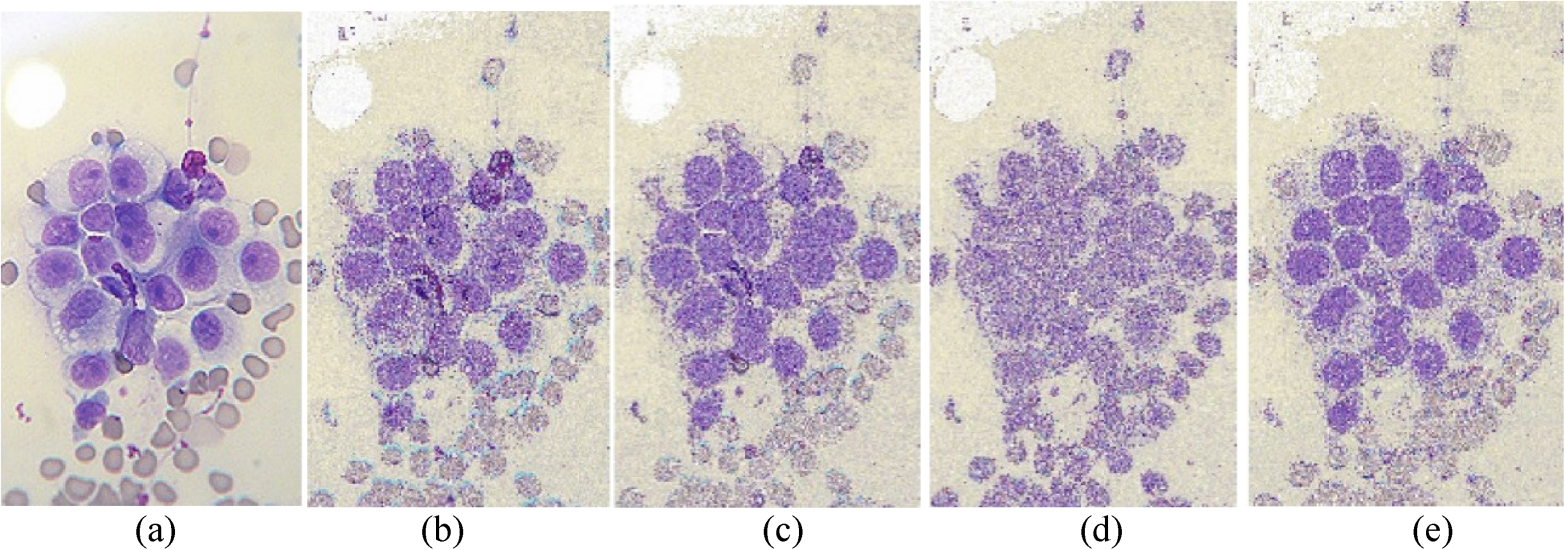
Comparison of *cytology* image. (a) Ground true (SSI = 1). Example of images obtained by: (b) FlexClustS [*n* = 1%*N*] (SSI = 0.2413), (c) FlexClustS [*n* = 2%*N*] (SSI = 0.2524), (d) Strategy III [*n* = 1%*N*] (SSI = 0.2351), and (e) Strategy III [*n* = 2%*N*] (SSI = 0.2388).

**Fig 10:**
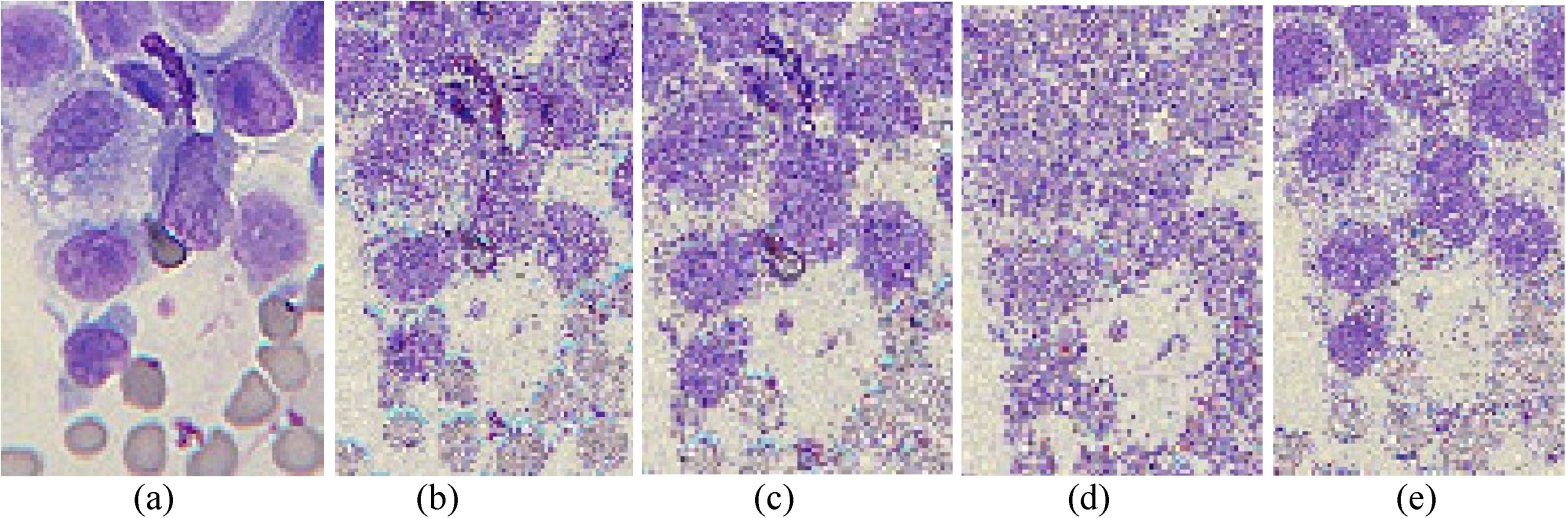
Close-up performance comparison of *cytology* image. (a) Ground truth (SSI = 1), (b) FlexClustS [*n* = 1%*N*] (SSI = 0.2413), (c) FlexClustS [*n* = 2%*N*] (SSI = 0.2524), (d) Strategy III [*n* = 1%*N*] (SSI = 0.2351), and (e) Strategy III [*n* = 2%*N*] (SSI = 0.2388).

**Fig 11:**
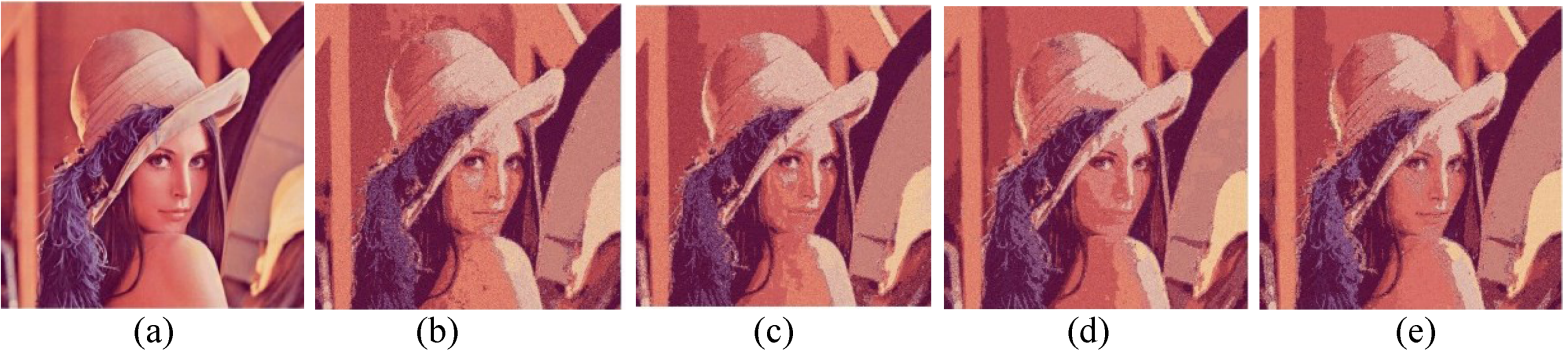
Comparison of *Lena* image. (a) Ground true (SSI = 1). Example of images obtained by: (b) FlexClustS [*n* = 0.5%*N*] (SSI = 0.6145), (c) FlexClustS [*n* = 1%*N*] (SSI = 0.6429), (d) Strategy III [*n* = 0.5%*N*] (SSI = 0.6708), and (e) Strategy III [*n* = 1%*N*] (SSI = 0.6633).

**Fig 12:**
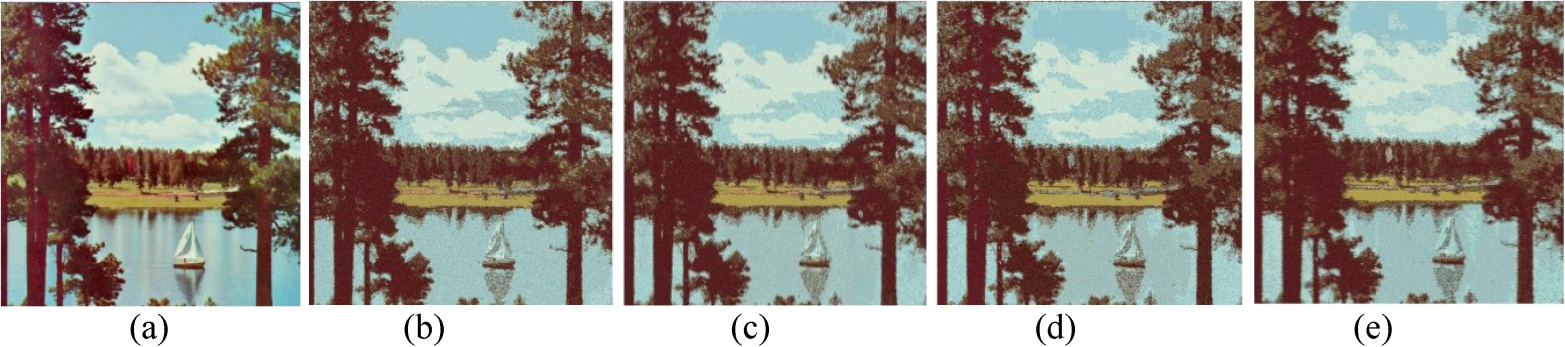
Comparison of *sailboat* image. (a) Ground true on (SSI = 1). Example of images obtained by: (b) FlexClustS [*n* = 0.5%*N*] (SSI = 0.6859), (c) FlexClustS [*n* = 1%*N*] (SSI = 0.6923), (d) Strategy III [*n* = 0.5%*N*] (SSI = 0.6739), and (e) Strategy III [*n* = 1%*N*] (SSI = 0.6702).

**Fig 13:**
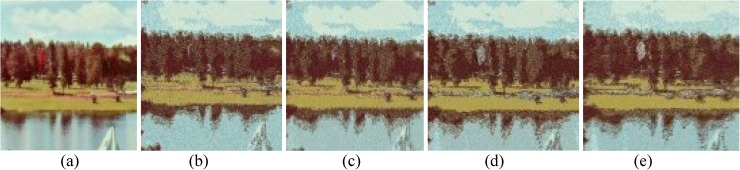
Close-up performance comparison of *sailboat* image. (a) Ground truth (SSI = 1), (b) FlexClustS [*n* = 0.5%*N*] (SSI = 0.6859), (c) FlexClustS [*n* = 1%*N*] (SSI = 0.6923), (d) Strategy III [*n* = 0.5%*N*] (0.6739), and (e) Strategy III [*n* = 1%*N*] (SSI = 0.6702).

**Fig 14:**
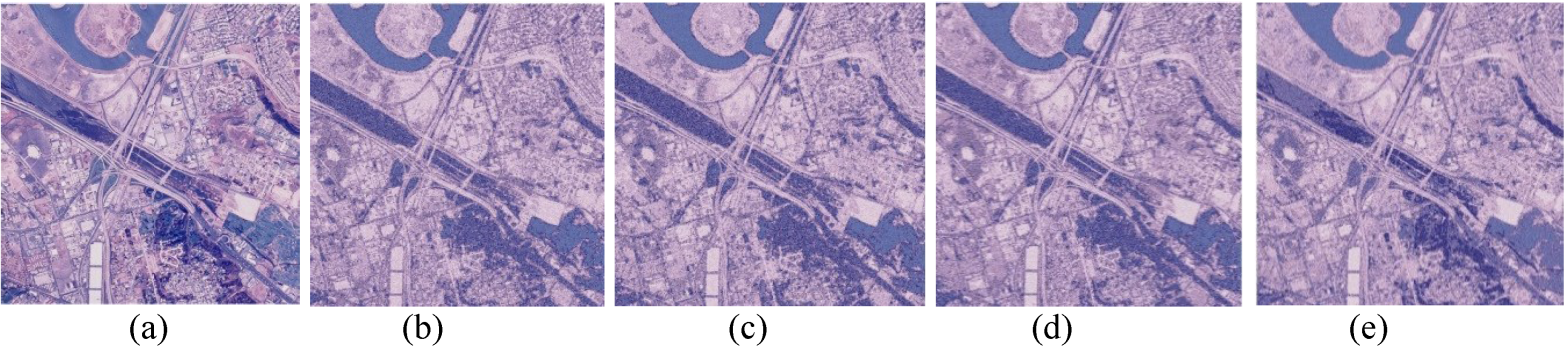
Comparison of *San Diego* image. (a) Ground true on (SSI = 1). Example of images obtained by: (b) FlexClustS [*n* = 0.5%*N*] (SSI = 0.6957), (c) FlexClustS [*n* = 1%*N*] (SSI = 0.7012), (d) Strategy III [*n* = 0.5%*N*] (SSI = 0.7179), and (e) Strategy III [*n* = 1%*N*] (SSI = 0.7240).

The performances of FlexClustS and Strategy III on the real image data are further visually evaluated. Samples of the reconstructed images are shown in Figs 7–14. Regardless of the sample sizes, FlexClustS performs better than Strategy III in recovering the small pixel clusters. For the *St Paulia* image, almost all the experiments of Strategy III missed out the small clusters of yellow flowers whereas FlexClustS reveals the yellow flowers in all the experiments, for examples see Figs 7 and 8. The pixels of the yellow flower are mistakenly assigned as the colour of the white background or leaves by the Strategy III algorithm. In the *cytology* image, FlexClustS recovers the small details of cell structure better than Strategy III. Examples of the results are shown in Figs 9 and 10. For *sailboat* images in Figs 12 and 13, it can be seen that the feature of the road is better preserved by FlexClustS compared to Strategy III. A considerable number of pixels of the road are mistakenly assigned as the colour of the sky or river.

Results of images processed through dividing image into blocks are shown in Table 4. FlexClustB demonstrates good overall performance. The SSIs of all the images by FlexClustB are higher than sufficient EM especially for *sailboat* (16x16), which are 0.8826 and 0.5506 respectively. It is interesting to note that the SSIs of FlexClustB by block are the highest in all the images even when compared to FlexClustS.

**Table 4:** Structural similarity indices (SSI) for images processed by dividing image into blocks.

Method	St Paulia		Cytology		Lena		Sailboat		San Diego	
	8x2 blocks	16x2 blocks	5x4 blocks	10x8 blocks	8x8 blocks	16x16 blocks	8x8 blocks	16x16 blocks	8x8 blocks	16x16 blocks
FlexclustB	**0.8318**	**0.8047**	**0.3169**	**0.3141**	**0.8332**	**0.8293**	**0.8687**	**0.8826**	**0.8423**	**0.7816**
Suffient EM	0.6315	0.7236	0.2111	0.1591	0.6786	0.6726	0.7115	0.5506	0.7435	0.6297

**Fig 15:**
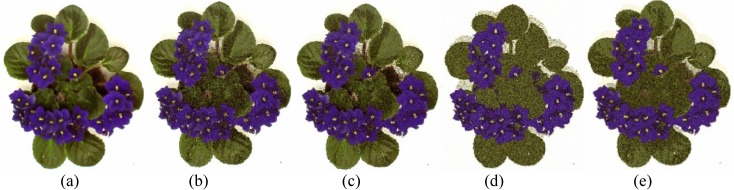
Comparison of *St Paulia* image. (a) Ground true (SSI = 1). Example of images obtained by FlexClustB on image division into: (b) 8x2 blocks (SSI = 0.8318), and (c) 16x2 blocks (SSI = 0.8047). Example of images obtained by sufficient EM on image division into: (d) 8x2 blocks, 60 sets of sufficient statistics per block (SSI = 0.6315), and (e) 16x2 blocks, 10 sets of sufficient statistics per block (SSI = 0.7236).

**Fig 16:**
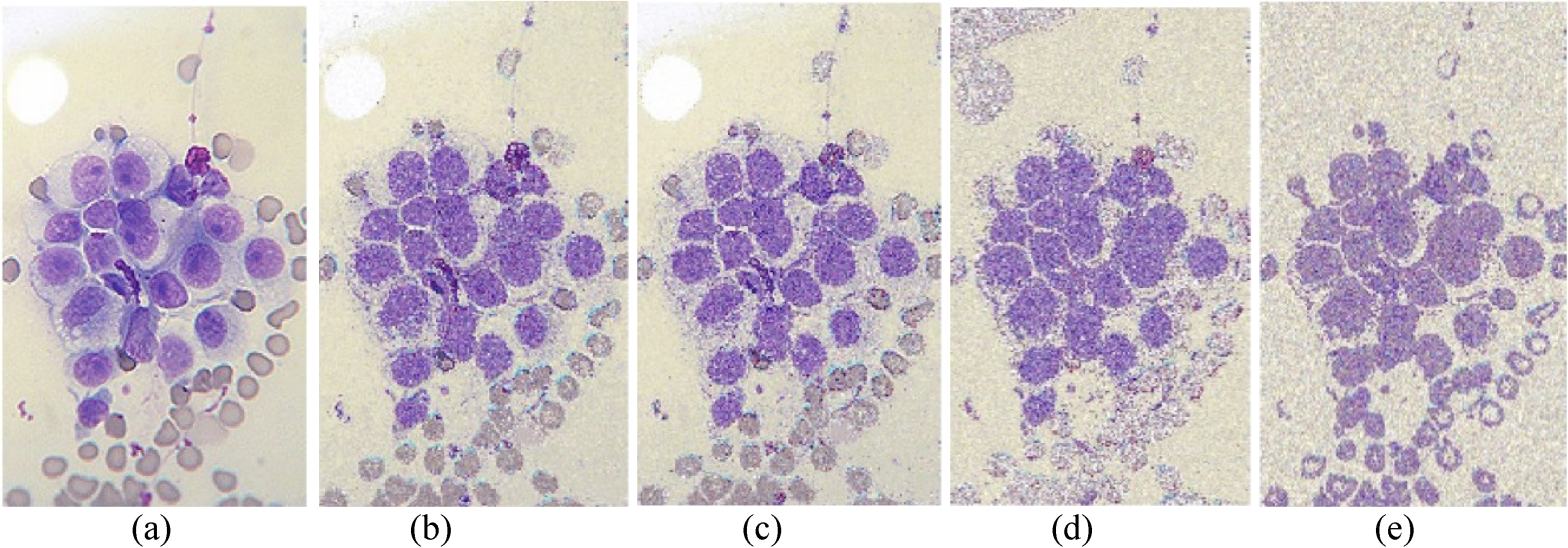
Comparison of *cytology* image. (a) Ground true (SSI = 1). Example of images obtained by FlexClustB on image division into: (b) 5x4 blocks (SSI = 0.3169), and (c) 10x8 blocks (SSI = 0.3141). Example of images obtained by sufficient EM on image division into: (d) 5x4 blocks, 60 sets of sufficient statistics per block (SSI = 0.2111), and (e) 10x8 blocks, 5 sets of sufficient statistics per block (SSI = 0.1591).

**Fig 17:**
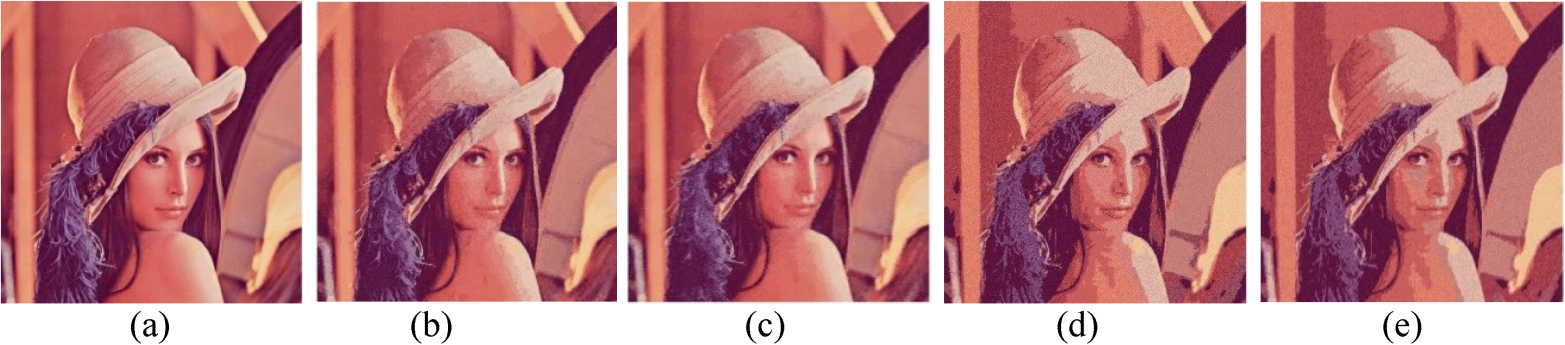
Comparison of *Lena* image. (a) Ground true (SSI = 1). Example of images obtained by FlexClustB on image division into: (b) 8x8 blocks (SSI = 0.8332), and (c) 16x16 blocks (SSI = 0.8293). Example of images obtained by sufficient EM on image division into: (d) 8x8 blocks, 65 sets of sufficient statistics per block (SSI = 0.6786), and (e) 16x16 blocks, 10 sets of sufficient statistics per block (SSI = 0.6726).

**Fig 18:**
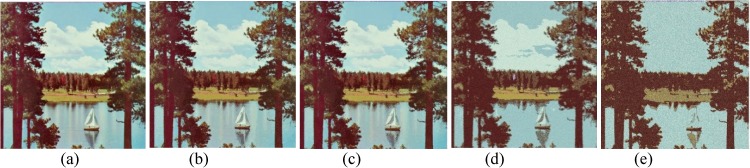
Comparison of *sailboat* image. (a) Ground true on (SSI = 1). Example of images obtained by FlexClustB on image division into: (b) 8x8 blocks (SSI = 0.8687), and (c) 16x16 blocks (SSI = 0.8826). Example of images obtained by sufficient EM on image division into: (d) 8x8 blocks, 65sets of sufficient statistics per block (SSI = 0.7115), and (e) 16x16 blocks, 3 sets of sufficient statistics per block (SSI = 0.5506).

**Fig 19:**
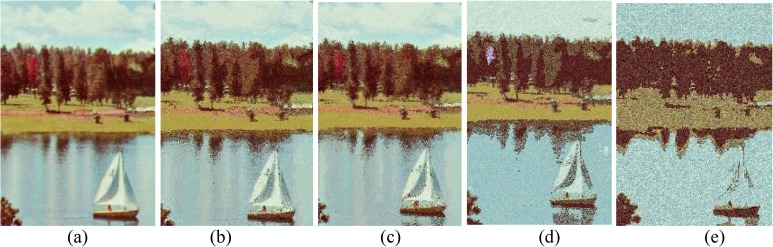
Close-up performance comparison of *sailboat* image. (a) Ground truth (SSI = 1), (b) FlexClulstB, 8x8 blocks (SSI = 0.8687), (c) FlexClust, 16x16 blocks (SSI = 0.8862), (d) sufficient EM, 8x8 blocks (SSI = 0.7115), and (e) sufficient EM, 16x16 blocks (SSI = 0.5506).

**Fig 20:**
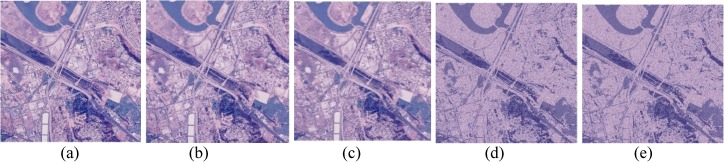
Comparison of *San Diego* image. (a) Ground true on (SSI = 1). Example of images obtained by FlexClustB on image division into: (b) 8x8 blocks (SSI = 0.8423), and (c) 16x16 blocks (SSI = 0.7816). Example of images obtained by sufficient EM on image division into: (d) 8x8 blocks, 65 sets of sufficient statistics per block (SSI = 0.7435), and (e) 16x16 blocks, 10 sets of sufficient statistics per block (SSI = 0.6297).

Examples of images processed by FlexClustB and sufficient EM are shown in Figs 15–20. When the images are assessed visually, it is found that regardless of the number of blocks the images are divided, FlexClustB recovers the images far better than the sufficient EM. Fig 15 shows that FlexClustB performs better than sufficient EM in identifying the leave structure especially in the middle part of the *St Paulia* flower. Fig 16 shows the details of the cell structure are better recovered by FlexClustB but sufficient EM misses the white spot on the left top of the image. In Fig 17, the images processed by FlexClustB show a significant gradient between the two parts of the hat, and *Lena*’s chin is separated from her shoulder as compared to sufficient EM. Figs 18 and 19 show that the red leaved tree which is located in the middle of the original image is recovered by FlexClustB but not by sufficient EM. Fig 20 shows that the satellite image processed by FlexClustB preserves the feature details far better than sufficient EM.

#### 4.6.2 Evaluation of number of clusters

Comparison between cluster numbers obtained by FlexClustS and Strategy III based on sample data, and between FlexClustB and sufficient EM based on division of image into blocks for the 5 images are summarized in Table 5. The results show that the numbers of clusters obtained by FlexClustS and FlexClustB are always more than Strategy III and sufficient EM, and thus produce better quality in image recovery. The results are consistent with the image segmentation results by [50] where insufficient number of clusters could lead to classification errors in image segmentation, and by Gaussian mixture model [51] which tends to describe similar structure in an image via the multiple components where each component represents different levels of contrast.

**Table 5:** Average number of clusters obtained for the 5 images.

Algorithm	*St Paulia*	*cytology*	*Lena*	*sailboat*	*San Diego*
Proposed FlexClustS [sample size 1]	61	25	40	37	25
Proposed FlexClustS [sample size 2]	69	22	46	50	35
Strategy III [sample size 1]	15	6	13	11	8
Strategy III [sample size 2]	15	6	14	13	9
Proposed FlexClustB [block size 1]	129	80	511	457	277
Proposed FlexClustB [block size 2]	197	87	1462	1152	336
Sufficient EM [block size 1]	18	5	21	16	5
Sufficient EM[block size 2]	20	24	15	21	3

Note: see Table 3 for sample size, and Table 4 for block sizes.

#### 4.6.3 Evaluation of computational time

With respect to computational time, FlexClustS and FlexCusterB are the slowest performer compared to the competitors for all the images, except for *cytology* with 16x2 blocks where FlexClustB needs 143.05 s as compared to sufficient EM which takes 147.19 s. From Tables 6 and 7, it again shows that FlexClust spents more time in processing samples or blocks which involves EM algorithm, but works faster when classifying multiple GMM MLEs for the entire data set. Its scan through and selection stage lasted from 0.34 s to 4.39 s for the improvement on GMM initialization. For Strategy III and sufficient EM, most of the computational time spent is also in the stage involving EM algorithm.

**Table 6:** Mean time (in secs) for image processing by sampling using FlexclustS and Stratgy III.

	FlexclustS				Strategy III		
Image	Scan through & select	Samples clustering	Components merging	Total	Sample clustering	EM to whole data set	Total
St Paulia (*n* = 1%*N*)	1.00	85.27	7.22	93.49	4.40	87.63	92.03
St Paulia (*n* = 2%*N*)	0.70	177.04	3.24	180.98	10.66	64.25	74.91
Cytology (*n* = 1%*N*)	0.61	111.89	1.88	114.38	5.30	8.49	13.80
Cytology (*n* = 2%*N*)	0.61	142.67	1.03	144.30	7.65	5.11	12.76
Lena (*n* = 0.5%*N*)	0.34	391.29	12.40	404.02	12.49	188.43	200.92
Lena (*n* = 1%*N*)	1.56	1401.03	7.14	1409.73	44.19	98.05	142.24
Sailboat (*n* = 0.5%*N*)	4.39	401.67	11.48	417.54	14.49	187.32	201.82
Sailboat (*n* = 1%*N*)	3.47	1462.57	7.65	1473.68	46.74	106.91	153.65
San Diego (*n* = 0.5%*N*)	3.60	1060.42	7.51	1071.53	17.70	59.14	76.84
San Diego (*n* = 1%*N*)	2.49	1111.82	4.94	1119.25	64.20	83.91	148.11

Note: *n* is the sample size, *N* is the image data size.

**Table 7:** Mean time (in secs) for image processing by block using FlexclustB and sufficient EM.

	FlexclustB				Sufficient EM		
Image	Scan through & select	Blocks clustering	Components merging	Total	Blocks compression	EM to sufficient statistics	Total
St Paulia (8x2 blocks)	0.73	1240.95	0.83	1242.51	2.36	96.34	98.70
St Paulia (16x2 blocks)	1.00	407.75	2.83	411.58	1.36	211.51	212.87
Cytology (5x4 blocks)	0.75	362.06	0.58	363.39	0.97	146.03	147.00
Cytology (10x8 blocks)	0.61	140.78	1.66	**143.05**	0.30	146.89	147.19
Lena (8x8 blocks)	4.10	2378.87	9.84	2392.81	5.94	987.88	993.82
Lena (16x16 blocks)	0.93	274.28	72.27	347.48	2.93	163.86	166.79
Sailboat (8x8 blocks)	4.13	2326.08	10.39	2340.60	5.93	749.93	755.86
Sailboat (16x16 blocks)	1.94	304.31	81.59	387.84	1.08	94.10	95.18
San Diego (8x8 blocks)	3.29	2279.95	8.76	2292.00	5.49	806.98	812.47
San Diego (16x16 blocks)	1.80	376.78	35.38	413.96	2.73	155.11	157.84

**Fig 21:**
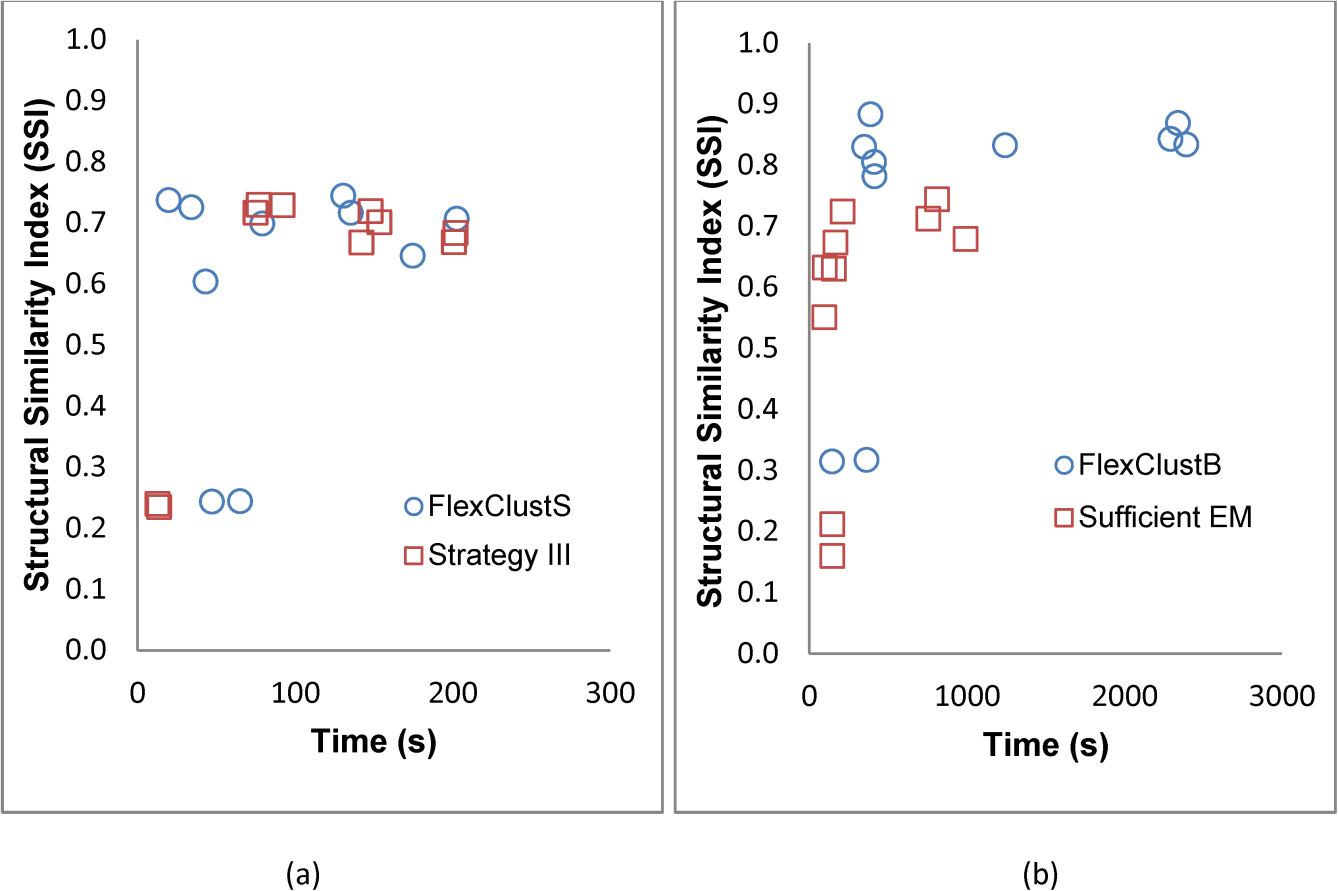
Comparison of structural similarity index (SSI) and computational time. (a) FlexClustS (based on 10%*N*) and Strategy III, (b) FlexClustB and sufficient EM.

Fig 21(A) shows the comparison of SSI and the computational time between FlexClustS processes based on 10% of the image data and Strategy III. It can be seen that in most cases, FlexClustS outperforms Strategy III in terms of computational time and at the same time does not trade off image quality. An extensive evaluation of the percentage of data that should be processed by FlexClustS in order to speed up the algorithm will be studied in future. In Fig 21(B), the SSI of FlexClustB is always higher than sufficient EM but at the cost of longer computational time.

## 5 Discussion and conclusion

In processing scaled down image either from sample data or blocks of divided image, the representation of partial image, the similarity measure and the domain adaptation are the three crucial issues to be addressed. The FlexClust algorithm is proposed to tackle these problems. FlexCust can be implemented in two ways either by dividing the image into multiple samples (FlexClustS) or blocks (FlexClustB).

Whatever methods used to represent the image, there is loss of information. The problem is even more challenging when working on partial image data. Small but important information tend to be missed out. It is important to address this issue especially in medical image as often there are only subtle differences in visual features between the normal and pathological images [13]. This paper tackles the problem with two approaches: (i) use detail preserving method for image representation, and (ii) recover small and useful information from multiple portions of full data. Most of the existing methods use distance based methods such as *k*-means to summarize the partial data and represent it in triplet of sufficient statistics [24–25, 52], which does not capture well of the image features if they are not spherical in shape. The results show that FlexClust enhances the possibility of the detection of small details of the image by using GMM with a scan through and selection procedure. The descriptors of local features by MLE of GMM captures features of different orientation, volume and size [20, 31]. In the case of sampling based method, it always leads to unstable results [1]. Although Strategy III chooses the best model from multiple tentative best models, the trained models are still based on the same sample data. The issue of lack of representativeness of sample has not been fully addressed. FlexClustS which incorporates multiple GMM clustering from multiple samples helps to alleviate the problem. The most unique part of FlexClustS is that it allows domain adaptation, where it recovers and incorporates the cluster that has not being identified in the previous samples as illustrated in the simulation study. The proposed domain adaptation makes use of only the GMM MLEs descriptors from the source and target domains. The existing domain adaptation techniques are performed mainly by reducing the difference between the distributions of the domains [53] or discovering a good feature representation across domains [54–55]. However, there is very limited work on domain adaptation for mixture model.

The choice of similarity measure is normally affected by how the image is represented and the type of descriptor used. FlexClust shows that by using MBF as a similarity measure to classify detail preserving descriptors of GMM MLEs can avoids loss of feature details. It is an improvement compared to [24–25], where their findings show that using GMM to classify descriptors (e.g. triplet of sufficient statistics) obtained from distance based clustering method (e.g. *k*-means) performs better than algorithms that use distance based clustering method for both classifying descriptors and producing descriptors of image representation [52]. From the results of the block based images, both FlexClustB and sufficient EM avoid the blocking artifact problem. This is the advantage of GMM based algorithm.

The results show that MBF works time effectively as a similarity measure, although relative to the other methods, FlexClustS and FlexClustB take longer computational time. However, the longer computational time is compensated by better quality image with a higher value of the SSI and better preservation of feature. It offers an alternative to medical imaging where good quality of image reconstruction is important and no loss of information can be tolerated [56]. Furthermore, it is worth noting that the second stage of FlexClust which involves the EM algorithm for multiple samples or blocks can be done independently. It leads to a substantial speed up by using parallel implementation on several processors [57].

Future work can be devoted to the generalization of the proposed algorithms to handle image with noise, and the optimal percentage of data that should be processed by FlexClustS in order to reduce the computational time in the second stage.

## Supporting information

S1 Fig Image of St Paulia. (JPG)

S2 Fig Image of cytology. (JPG)
