# Supplementary figures and images for: Similarity measure and domain adaptation in multiple mixture model clustering: An application to image processing
Source: PLoS One. 2017 Jul 7;12(7):e0180307. doi: 10.1371/journal.pone.0180307 (PMC5501543; doi:10.1371/journal.pone.0180307)

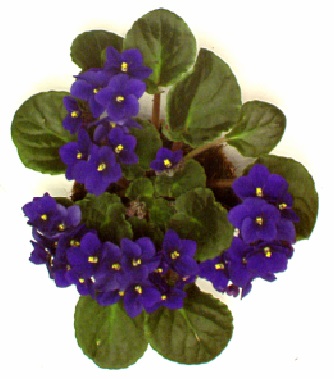

Supplement: S1 Fig — (JPG) [file pone.0180307.s001.jpg]

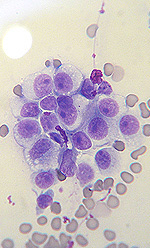

Supplement: S2 Fig — (JPG) [file pone.0180307.s002.jpg]
